# Presymptomatic change in microRNAs modulates Tau pathology

**DOI:** 10.1038/s41598-018-27527-6

**Published:** 2018-06-18

**Authors:** Salil Sharma, Ines Khadimallah, Adam Williamson Corya, Yousuf Omar Ali, Xi Rao, Yunlong Liu, Hui-Chen Lu

**Affiliations:** 1 Department of Psychological and Brain Sciences, the Linda and Jack Gill Center for Bimolecular Sciences, Indiana University, Bloomington, IN 47405 USA; 2 Department of Fundamental Neurosciences (DNF), University of Lausanne (UNIL), Lausanne, Switzerland; 3 Medical and Molecular Genetics, Indiana University School of Medicine, Indianapolis, Indiana 46202 USA

## Abstract

MicroRNAs (miRs) are 18~23 nucleotides long non-coding RNAs that regulate gene expression. To explore whether miR alterations in tauopathy contribute to pathological conditions, we first determined which hippocampal miRs are altered at the presymptomatic and symptomatic stages of tauopathy using rTg4510 mice (Tau mice), a well-characterized tauopathy model. miR-RNA pairing analysis using QIAGEN Ingenuity Pathway Analysis (IPA) revealed 401 genes that can be regulated by 71 miRs altered in Tau hippocampi at the presymptomatic stage. Among several miRs confirmed with real-time qPCR, miR142 (−3p and −5p) in Tau hippocampi were significantly upregulated by two-weeks of age and onward. Transcriptome studies by RNAseq and IPA revealed several overlapping biological and disease associated pathways affected by either Tau or miR142 overexpression, including Signal Transducer and Activator of Transcription 3 (*Stat3*) and Tumor Necrosis Factor Receptor 2 (*Tnfr2*) signaling pathways. Similar to what was observed in Tau brains, overexpressing miR142 in wildtype cortical neurons augments mRNA levels of Glial Fibrillary Acidic Protein (*Gfap*) and Colony Stimulating Factor 1 (*Csf*1), accompanied by a significant increase in microglia and reactive astrocyte numbers. Taken together, our study suggests that miR alterations by Tau overexpression may contribute to the neuroinflammation observed in Tau brains.

## Introduction

MicroRNAs (miRs) constitute an abundant class of non-coding RNA molecules that are endogenously expressed in multicellular organisms. They are 18–23 nucleotides long, and often regulate gene expression by mRNA degradation or translational repression^1,2^. Many miRs are enriched in brain, and contribute to brain development, neural plasticity and neuroprotection^3–8^. The levels of many miRs are altered in neurodegenerative diseases^9–12^. For example, Lau *et al*. identified 35 miRs altered in the prefrontal cortex of late-onset Alzheimer’s Disease (LOAD) patients^13^. Interestingly, deleting Dicer, a critical enzyme involved in miR synthesis, in adult brain results in Tau hyperphosphorylation and neuronal loss, the pathological phenotypes associated with tauopathy, manifested as deposition of intracellular neurofibrillary tangles^11^. However, it is unclear whether miR changes are already present at the presymptomatic stages of neurodegenerative diseases, and if such miR changes can directly impact biological pathways that may subsequently lead to neurodegeneration.

Tauopathies include a wide range of neurodegenerative diseases such as progressive supranuclear palsy, Pick’s disease, parkinsonism linked to chromosome 17 (FTDP-17) and AD^14–17^. Previously, miRs have been identified as modulators of Tau, and contribute to Tau pathology^18–21^. Santa-Maria *et al*. showed that Tau is one of the direct targets of miR219^22^. MiR219 downregulation aggravated Tau induced toxicity, whereas its overexpression alleviated the toxic effects. Therefore, miR219 expression may modulate Tau toxicity by post-transcriptionally repressing Tau expression. In AD brains, miR132-3p levels are mainly decreased in neurons enriched with hyper-phosphorylated Tau^13^. Several genes implicated in Tau network, including the transcription factor (TF) FOXO1a, were identified as miR132-3p targets by in silico methods^13^. Knocking out the miR132/212 cluster results in an increase in Tau phosphorylation and aggregation. Interestingly, in an AD mouse model, miR132 treatment rescued memory deficits^23^. Consistent with previous studies, genome-wide studies using 39 AD and 25 control brains found significant downregulation of miR132/212 cluster in AD temporal cortex (TC)^24^. It is unclear how altered miR levels lead to the abnormal regulation of biological pathways that contribute to Tau pathology. The mechanistic insights on how miRs contribute to Tau pathology will provide new insights governing disease outcomes.

In this study, we aimed to identify RNA changes contributing to tauopathy. We first determined both miR and mRNA changes at presymptomatic stage of tauopathy. Second, miR-RNA pairing analysis was conducted to identify putative miR targets. Third, we overexpressed miR142, one of the identified miRs upregulated in tauopathy, in wildtype brains to examine its impact on gene expression. Fourth, bioinformatics analysis revealed several overlapping gene transcriptional changes as well as canonical and disease-associated pathways between Tau and miR142 overexpression. In summary, miR changes at an early stage of tauopathy are likely to contribute to disease progression.

## Results

### Age-dependent miR expression changes in Tau hippocampi

The rTg4510 mouse line (abbreviated as Tau mice for simplicity) has been widely used and characterized as a tauopathy mouse model^25–27^. These mice overexpress human Tau that carries the P301L mutation identified in frontotemporal dementia with Parkinsonism on chromosome 17 (FTDP-17) under the control of CAMKIIα-tTA^26^. Forebrain-Tau(P301L) expression starts after birth and peaks at around 2 months (2 m) of age^26,28^. Signs of neurodegeneration become detectable at 2.5 m. Tau tangles are observed in the cortex after 4 m and in the hippocampus after 5.5 m accompanied with severe age dependent cognitive decline and neuronal loss^28^. To determine whether Tau(P301L) overexpression alters miR levels, we employed RNAseq (Illumina GAIIx platform with raw reads of 40 nucleotides) to assess miR changes at two time points: An early presymptomatic stage when no obvious abnormalities are detected (2 m) (control, n = 3; Tau, n = 3; Supplementary Table 1); and a late symptomatic stage when severe Tau pathology and neuronal loss are evident (6 m) (control, n = 2; Tau, n = 4; Supplementary Table 1).

**Figure 1:**
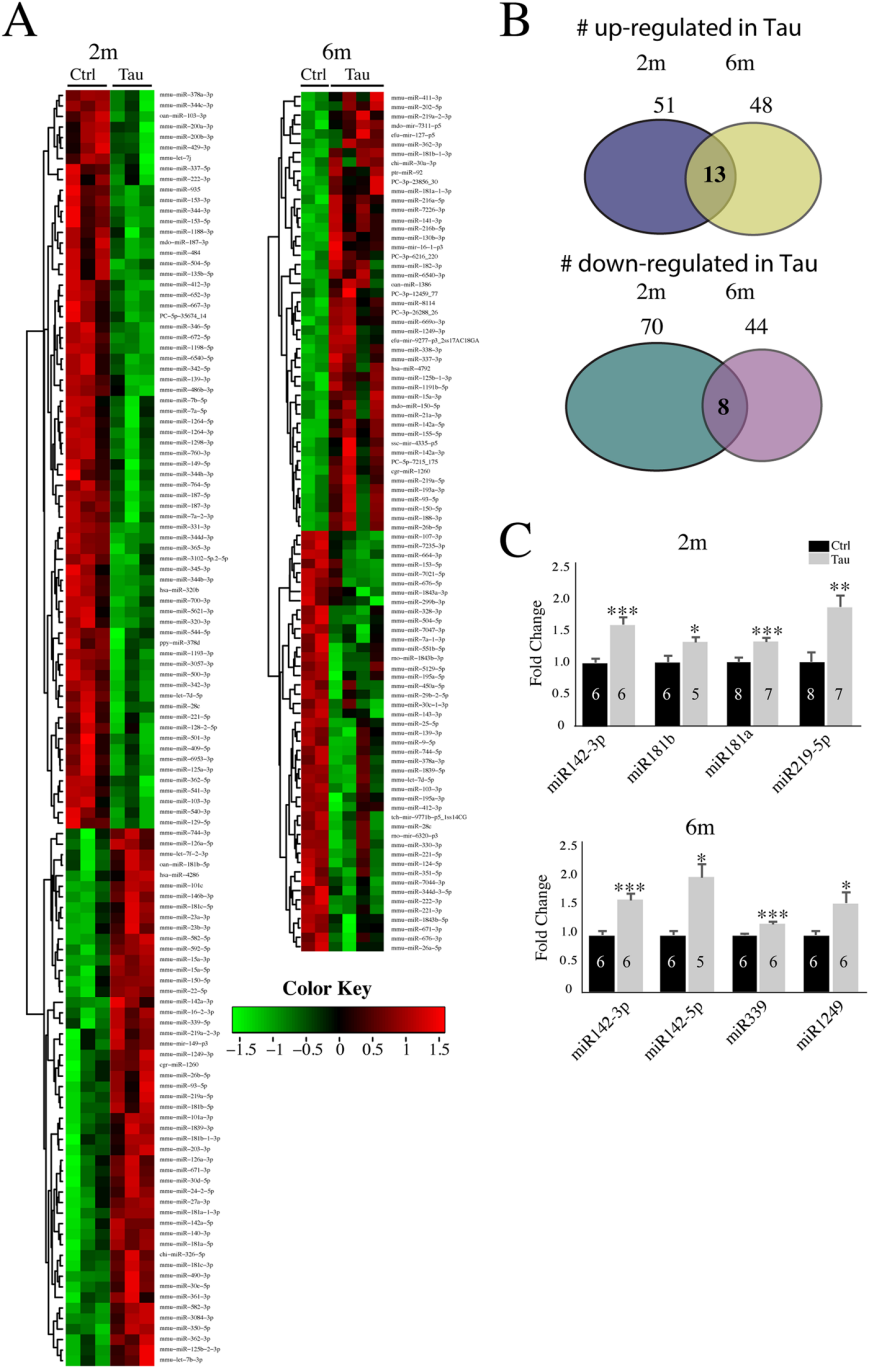
miRNA sequencing reveals alteration in miR expression at 2 m and 6 m in Tau hippocampus. (**A**) Heat maps show miRs that are differentially expressed between ctrl and Tau hippocampi at 2 m (ctrl, n = 3; Tau, n = 3) and 6 m (ctrl, n = 2; Tau, n = 4). (**B**) Summary for the number of up- and down-regulated miRs between 2 m and 6 m hippocampi. (**C**) Summary of qPCR data on selected miRs in 2 m and 6 m hippocampi. Error bars represent SEM. *p < 0.05, **p < 0.01, ***p < 0.001.

Differential expression analysis for known miRs between control and Tau hippocampi (depicted by heat maps in Fig. 1A) shows that: 121 miRs are significantly changed at 2 m (P < 0.05) with 51 upregulated and 70 downregulated in Tau hippocampi; 92 miRs are significantly changed (P < 0.05) at 6 m with 48 upregulated and 44 downregulated in Tau hippocampi (Fig. 1B). Among these miR changes, 21 miRs are altered in the same direction for both 2 m and 6 m (13 upregulated and 8 downregulated; a complete list of the differentially expressed annotated miRs is provided in Supplementary Tables 2 and 3 and GSE106967). Selected miRs were experimentally validated by qPCR analysis. For 2 m, miR142-3p, miR181b, miR181a (also known as miR213) and miR219-5p were validated; and for 6 m, validation was performed for miR142-3p, miR142-5p, miR339 and miR1249 (Fig. 1C).

### miR-RNA pairing analysis to identify putative miR targets and pathways in Tau hippocampi at a presymptomatic stage

**Figure 2:**
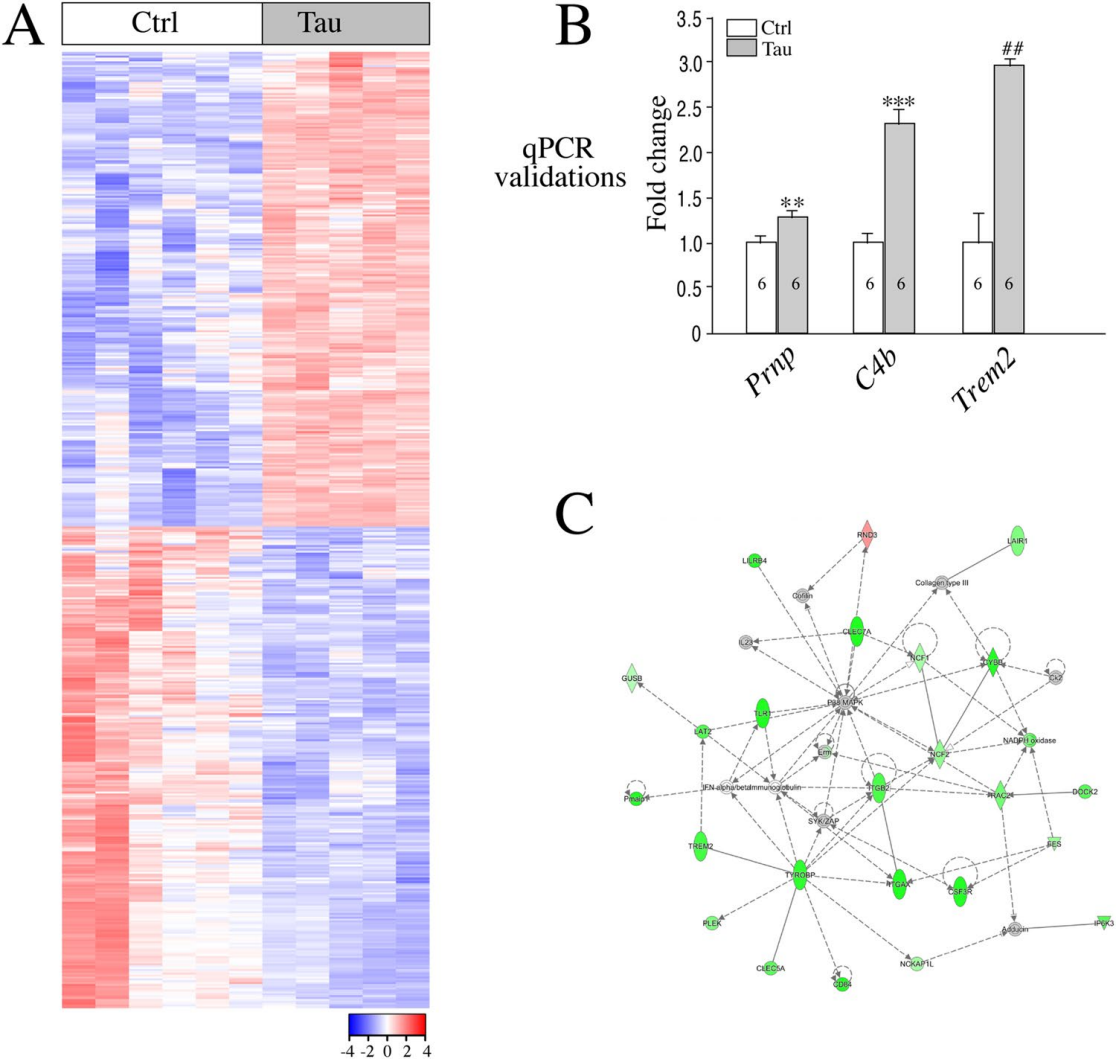
mRNA sequencing reveals transcriptome changes in 2 m Tau hippocampus. (**A**) Heat map shows genes that are significantly altered by Tau OE in 2 m hippocampi (n = 6 ctrl; 5 Tau). (**B**) qPCR data summary for normalized *Prnp*, *C4b*, and *Trem2* mRNA levels in 2 m hippocampi. (**C**) Inflammatory Response, Cell-To-Cell Signaling and Interaction, Cellular Function and Maintenance network is one of the top networks altered in 2 m Tau hippocampi.

In order to correlate the global effect on gene expression that, in part, is contributed by miR alterations in the Tau hippocampus, we performed mRNA sequencing with 2 m hippocampi. A multi-dimensional scaling (MDS) plot, assessing the similarities/differences among the expression profiles, shows clear separation between control and Tau hippocampi (control, n = 5; Tau, n = 6; Supplementary Fig. 2). The differential expression (DE) analysis identified 254 upregulated and 258 downregulated genes (FDR < 0.05) (Fig. 2A; Supplementary Table 4; GSE107183). qPCR experiments validated the differential expression levels of the following genes: Prion Protein (*Prnp*), Complement C4B (*C4b*) and Triggering Receptor Expressed on Myeloid Cells 2 (*Trem2*) (Fig. 2B).

Ingenuity Pathway Analysis (IPA) (Ingenuity H Systems, www.ingenuity.com) was then used for network analysis to correlate the observed transcriptomic changes to underlying Canonical Pathways, Transcriptomic Networks, and Disease Biofunctions (Supplementary Table 5). The top canonical signaling pathway revealed by IPA analysis corresponds to “Neuroinflammation Signaling Pathway”, which includes Interleukin 6 Receptor *(IL6R)*, *Trem2 and* Colony-Stimulating Factor 1 Receptor *(Csf1r)* as key genes (Supplementary Table 5). Inflammatory Response, Cell-To-Cell Signaling and Interaction, Cellular Function and Maintenance network was identified as one of the top 5 transcriptomic networks (Fig. 2C). This network includes *Csf3r*, Interferon alpha/beta (*Ifn-alpha/beta*) and *Trem2*, key genes involved in inflammation.

Next, we conducted miR-mRNA pairing analysis to assess the potential transcriptome changes resulting from miR-alterations in 2 m Tau hippocampi. Specifically, IPA analysis was conducted to examine the overlap between miR-targets, identified by IPA’s “microRNA Target Filter” feature, and differentially changed mRNAs from 2 m Tau hippocampi, identified by RNA sequencing. IPA “microRNA Target Filter” predicts miR targets with data collected in TargetScan’s human predicted target dataset, and experimentally validated targets from TarBase. This pairing analysis found 72 miRNAs pair with 401 gene targets (Supplementary Table 6). Pathway analysis of these overlapping targets revealed that “Neuroinflammation Signaling Pathway” is the top canonical pathway, “Neurological Disease” is the top disease in disease and biofunctions, and “Cell-To-Cell Signaling and Interaction, Cellular Movement, Hematological System Development and Function” is the top transcriptomic network (complete lists of pathways are shown in Supplementary Table 7). In addition to “Neuroinflammation Signaling Pathway”, several other canonical pathways involved in inflammation and immune response were also altered, such as “Fcγ Receptor-mediated Phagocytosis in Macrophages and Monocytes” and “Role of NFAT in Regulation of the Immune Response”.

### MiR142 is significantly induced in the hippocampus and cortex of Tau mice

MiR142-3p has been shown to regulate neuroinflammation associated with Multiple Sclerosis^29^. Upregulation of both miR142-3p and -5p was reported in the prefrontal cortex of LOAD patients^13^. miR181a and miR181b are involved in alteration of synaptic plasticity associated with neuropathology in a murine model of AD^30^. Santa-Maria *et al*. showed that miR219 represses Tau synthesis at the post transcriptional level^22^. Thus, we prioritized miR142-3p, miR142-5p, miR181a, miR181b, miR219-3p and miR219-5p to examine their temporal expression profiles in control and Tau hippocampi.

**Figure 3:**
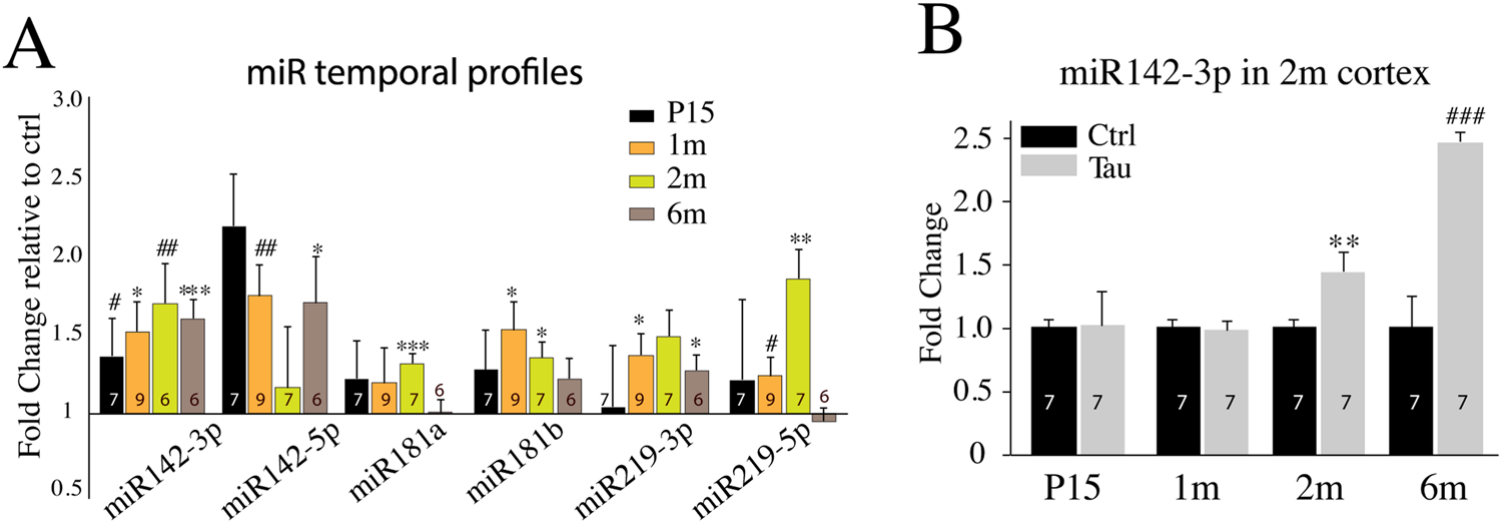
MiR142 levels are upregulated in Tau brains. (**A**) qPCR data show the time course for the level changes of selected miRs in Tau hippocampi. (**B**) qPCR data show miR142-3p is up-regulated in Tau cortex at 2 m and 6 m.

In Tau mice, Tau(P301L) is driven by CaMKIIα-tTA and begins to express around postnatal day 7^28^. To examine whether miR changes occurred shortly after Tau(P301L) overexpression, we performed qPCR to determine the temporal expression profiles of miRs in control mice without CaMKIIα-tTA or hTau(P301L) transgenes and Tau hippocampi at postnatal day 15, 1 m, 2 m and 6 m (Fig. 3A). The expression of miR142-3p was induced at postnatal day 15 and persisted until 6 m. Thus, the upregulation pattern of miR142-3p correlates well with the time course of Tau(P301L) overexpression (OE). The upregulation of miR142-5p, miR181b, miR219-3p and miR219-5p became significant at 1 m (Fig. 3A). Tau mice carry one copy of CaMKIIα-tTA and one copy of hTau transgene. Previous reports have shown that CaMKIIα-tTA mice also exhibit neurodegeneration and behavioral phenotypes^31,32^. To determine whether a single CamKIIα-tTA or hTau transgene OE can cause miR142-3P upregulation, we conducted qPCR experiments with 2 m hippocampi from CamKIIα-tTA-only and hTau transgene-only mice. No significant differences in miR142-3p levels were observed between control mice containing neither transgene and control mice with only one of the two transgenes, confirming that CaMKII-tTA transgene alone has no effect on miR142-3p expression (Supplementary Fig. 3). In addition to hippocampus, we found that miR142-3p expression is induced in Tau cortex at 2 m and 6 m (Fig. 3B). *In situ* hybridization (ISH) with anti-miR142-5p DNA probe in combination with immunostaining (IHC) using NeuN (recognizing neurons) and Iba-1 (recognizing microglia) antibodies suggest that miR142 upregulation is likely to occur in both neurons and microglia (Supplementary Fig. 4).

### Neuronal miR142 overexpression in developing cortex of wildtype brain leads to many transcriptional changes

**Figure 4:**
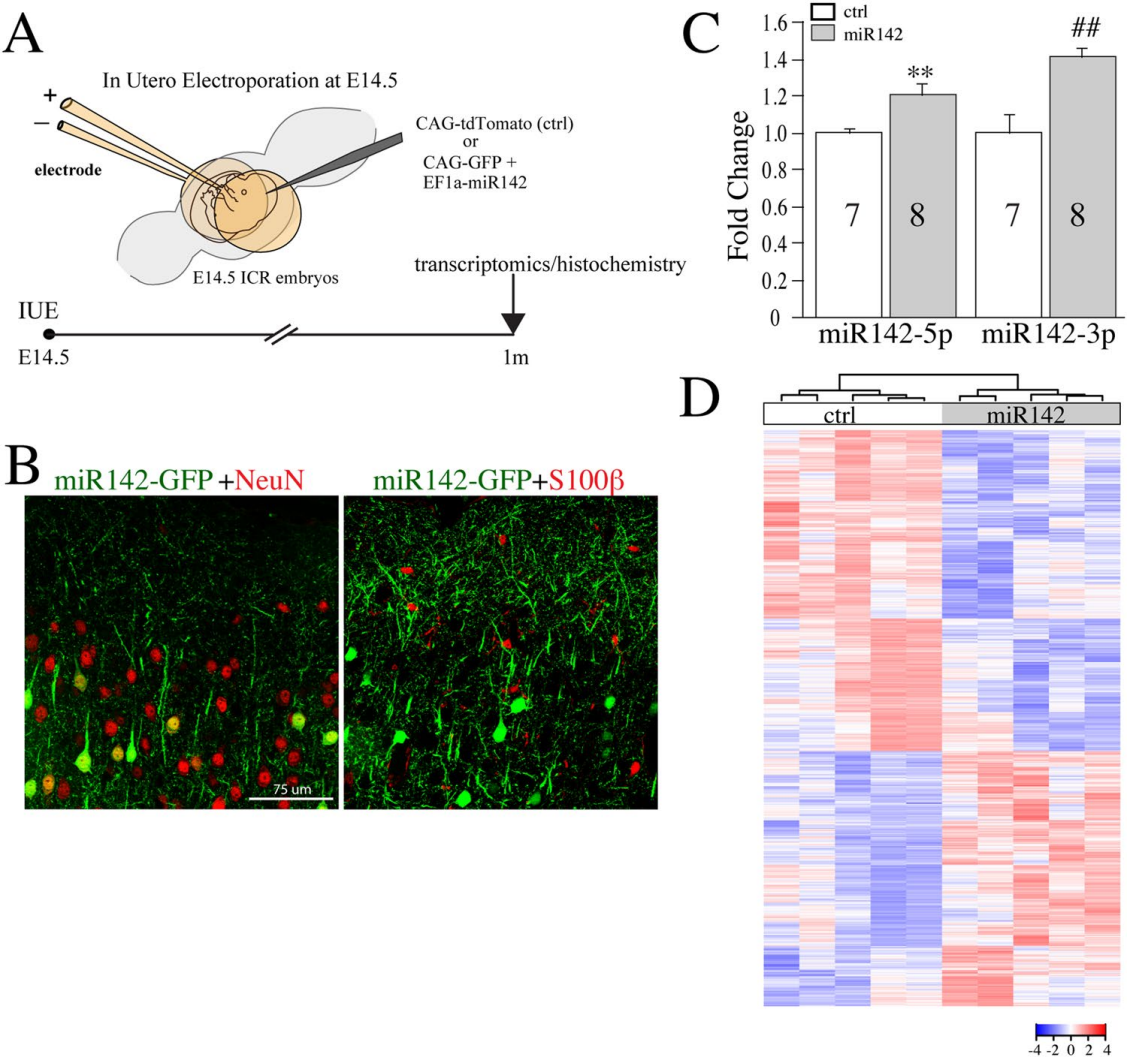
MiR142-OE in cortical neurons *in vivo* results in substantial transcriptome changes in cortex of wildtype mice. (**A**) Schematics of in-utero electroporation (IUE) technique used to overexpress miR142. (**B**) Localization of miR142-GFP (green) in neurons (red) but not in astrocytes (red) after IUE. (**C**) qPCR summary for normalized levels of miR142 (-3p and -5p) in the cortex of 1 m wildtype mice. (**D**) Heat map shows genes that are significantly altered by miR142-OE in wildtype cortex at 1 m (ctrl, n = 5; miR142OE, n = 5).

The early and persistent miR142 upregulation in Tau hippocampi, and its documented involvement in the inflammatory axis^29,33–36^ prompted us to examine its direct impacts on gene transcription. The in utero electroporation (IUE) technique is a widely used technique to deliver gene expression constructs into cortical neurons at late embryonic stages^37,38^. Here we employed IUE to introduce miR142 or control expression cassette into cortical neurons to assess the impact of miR142-OE on the cortical transcriptome at 1 m. Specifically, we electroporated miR142 together with GFP-reporter or with a tdTomato-reporter expression cassette alone (served as control) into the developing cortical plate of wildtype embryos at embryonic day 14.5 (E14.5) and let these embryos developed to term for birth (Fig. 4A). Using double-immunostaining with 1 m miR142-OE brains, we found that the majority of GFP-positive cells were also NeuN-positive (neuronal marker)^39^ but not S100β-positive (astrocyte marker)^40^ (Fig. 4B). qPCR analysis revealed a modest but significant increase in miR142 (−5p, 1.2 fold; and −3p, 1.4 fold) expression in the cortex prepared from miR142OE when compared with control cortices (Fig. 4C).

To assess transcriptome changes induced by miR142-OE in cortical neurons at 1 m, we performed mRNA sequencing on miR142-OE (GFP-positive) versus control (tdTomato-positive) cortices. The MDS plot obtained after mRNA-seq shows clear separation between miR142-OE and control samples (n = 5 per group, Supplementary Fig. 5). miR142-OE caused significant downregulation of 641 genes and upregulation of 501 genes (FDR < 0.05) (Fig. 4D, Supplementary Table 8). The gene network and pathway analysis showed that the top transcriptomic network altered by miR142-OE is “Cellular Assembly and Organization, Cellular Function and Maintenance, Cellular Movement” (Supplementary Table 9; GSE107167). The top canonical pathways, and top diseases and biofunctions altered by miR142-OE were also analyzed (Supplementary Table 10). These data provide the first *in vivo* demonstration that miR142-OE in cortical neurons can lead to substantial transcriptional changes.

### Overlapping gene expression changes between miR142 and Tau overexpression

**Figure 5:**
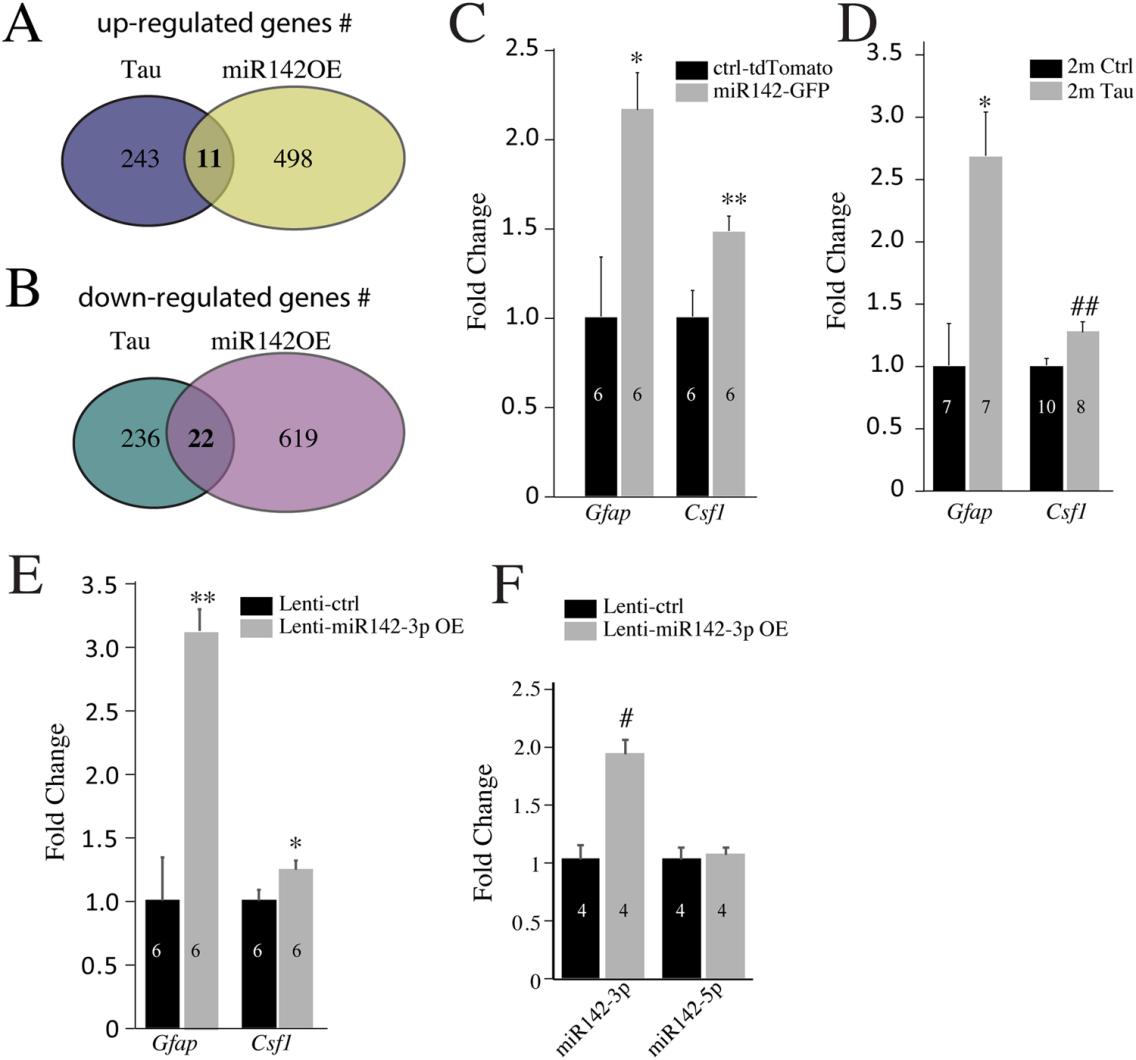
*Gfap* and *Csf1* mRNAs are up-regulated in both Tau and miR142-OE mice. (**A**,**B**) Overlap of up- and down-regulated genes between Tau hippocampi and miR142-OE cortices. (**C**,**D**,**E**) QPCR validation of induction of *Gfap* and *Csf1* genes in miR142-OE cortex, Tau hippocampus, and miR142-3p OE in primary cortical neuronal cultures. (**F**) qPCR validation for the up-regulation in miR142-3p levels in primary cortical neurons transduced with miR142-3p lentivirus.

Comparison of gene changes in miR142-OE and Tau-OE, based on the mRNA-seq data presented above, revealed 11 upregulated and 22 downregulated genes common between two datasets (Fig. 5A,B; Table 1). Among the upregulated genes, both *Gfap* and *Csf1* are known to play key roles in inflammation and gliosis^41–43^. GFAP is considered a marker of astrogliosis^44,45^ while CSF1 controls the development and functions of macrophages, such as microglia^46^. Mutations in the *Csf1r* gene are associated with progressive dementia^46^ and inhibiting CSF1R increases disease severity in animal models of AD^47^. We validated these changes using qPCR in both miR142-OE cortex (Fig. 5C) and Tau OE hippocampus (Fig. 5D). Interestingly, applying lentivirus vector carrying miR142-3p expressing cassette *in-vitro* to (DIV7) primary cortical neurons also led to *Gfap* and *Csf1* upregulation at DIV11 in addition to the increase of miR142-3P (Fig. 5E,F).

**Table 1:** List of similarly regulated genes that overlap between Tau and miR142-OE mice determined by IPA analysis.

Up-regulated genes	Up-regulated gene names	Down-regulated genes	Down-regulated gene names
Gfap	Glial Fibrillary Acidic Protein	Esyt2	Extended Synaptotagmin 2
Islr2	Immunoglobulin Superfamily Containing Leucine Rich Repeat 2	Grm4	Glutamate Metabotropic Receptor 4
Csf1	Colony Stimulating Factor 1	Scn4b	Sodium Voltage-Gated Channel Beta Subunit 4
Wnt7b	Wnt Family Member 7B	Pcp4l1	Purkinje Cell Protein 4 Like 1
Trpv2	Transient Receptor Potential Cation Channel Subfamily V Member 2	Nexn	Nexilin F-Actin Binding Protein
Anxa7	Annexin A7	Kcnh4	Potassium Voltage-Gated Channel Subfamily H Member 4
Fgf5	Fibroblast Growth Factor 5	Rasd2	RASD Family Member 2
Tmem178	Transmembrane Protein 178 A	Unc13c	Unc-13 Homolog C
Rxfp1	Relaxin/Insulin Like Family Peptide Receptor 1	Calb1	Calbindin 1
Pde1a	Phosphodiesterase 1 A	Pdzd2	PDZ Domain Containing 2
Clcnka	Chloride Voltage-Gated Channel Ka	Myh7	Myosin Heavy Chain 7
		Strip2	Striatin Interacting Protein 2
		Pde7b	Phosphodiesterase 7B
		Dach1	Dachshund Family Transcription Factor 1
		Stc1	Stanniocalcin 1
		Pou3f4	POU Class 3 Homeobox 4
		Ptprv	Protein Tyrosine Phosphatase, Receptor Type V
		Slc32a1	Solute Carrier Family 32 Member 1
		Per3	Period Circadian Clock 3
		Scarb1	Scavenger Receptor Class B Member 1
		Kcnj2	Potassium Voltage-Gated Channel Subfamily J Member 2
		Gpr88	G Protein-Coupled Receptor 88

**Figure 6:**
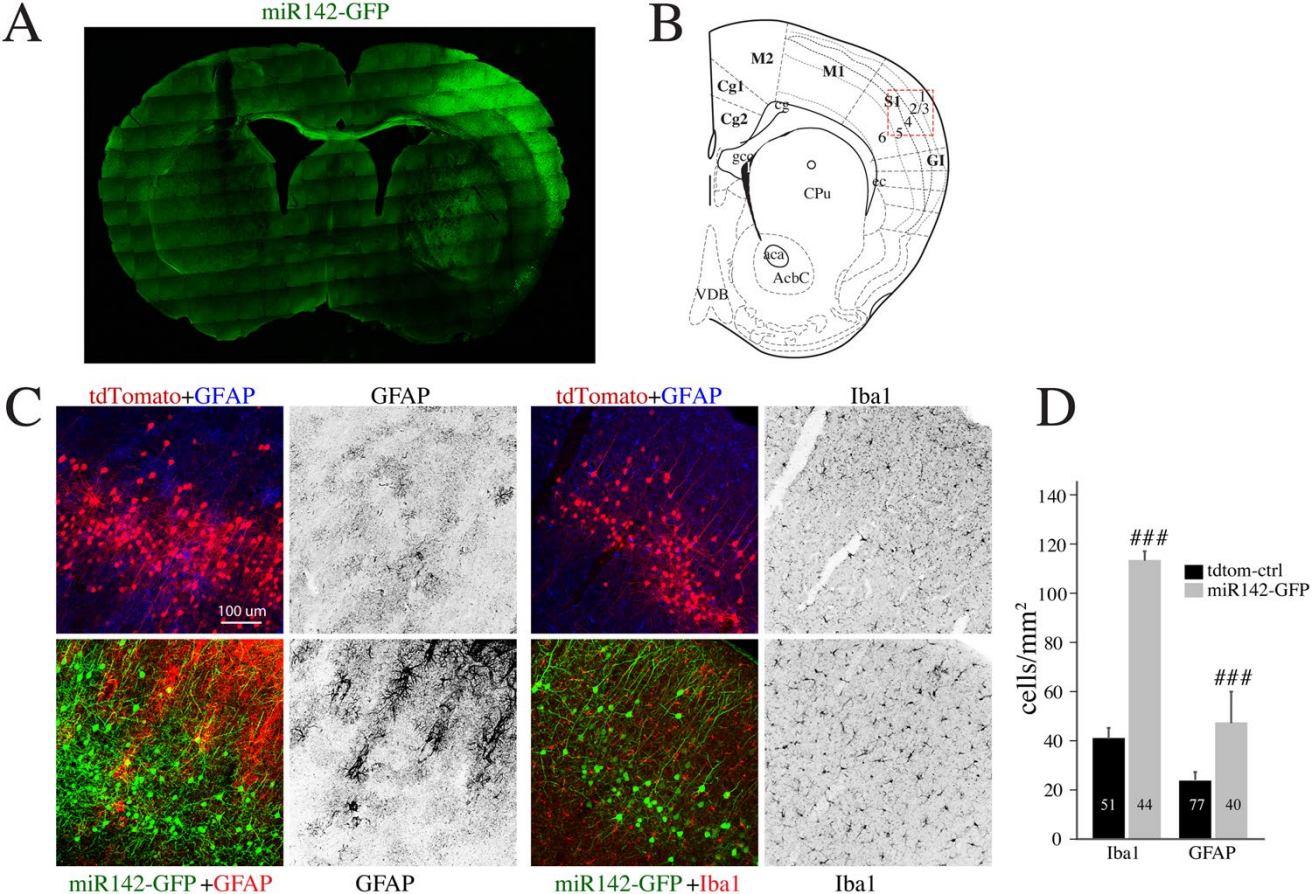
MiR142-OE results in increase in microglia and astrocytes. (**A**) Whole brain scan shows the location of miR142-GFP OE in 1 m old wildtype mice. (**B**) Specific location of miR142 and td-tomato control OE in the mouse brain map. (**C**) Increase of microglia (*Iba1* staining) and astrocytes (*Gfap* staining) in the cortex of miR142-OE vs tdTomato controls shown by immunofluorescence. (**D**) Quantification of *Iba1* positive microglia and *Gfap* positive astrocytes shown in (**C**).

To assess whether miR142-OE in cortical neurons could lead to infiltration or proliferation of microglia and astrocytes in miR142-OE cortex, we performed immunostaining using Iba-1 and GFAP antibodies. Significant increases in Iba-1 positive microglia and GFAP-positive reactive astrocytes were observed in miR142-OE cortical regions compared to tdTomato controls (Fig. 6A–D). This observation suggests that miR142-OE in neurons could elicit inflammatory signals that lead to an increase in microglia and reactive astrocytes numbers.

### MiR142-OE exerts similar effect as Tau-OE on biological and disease networks

**Figure 7:**
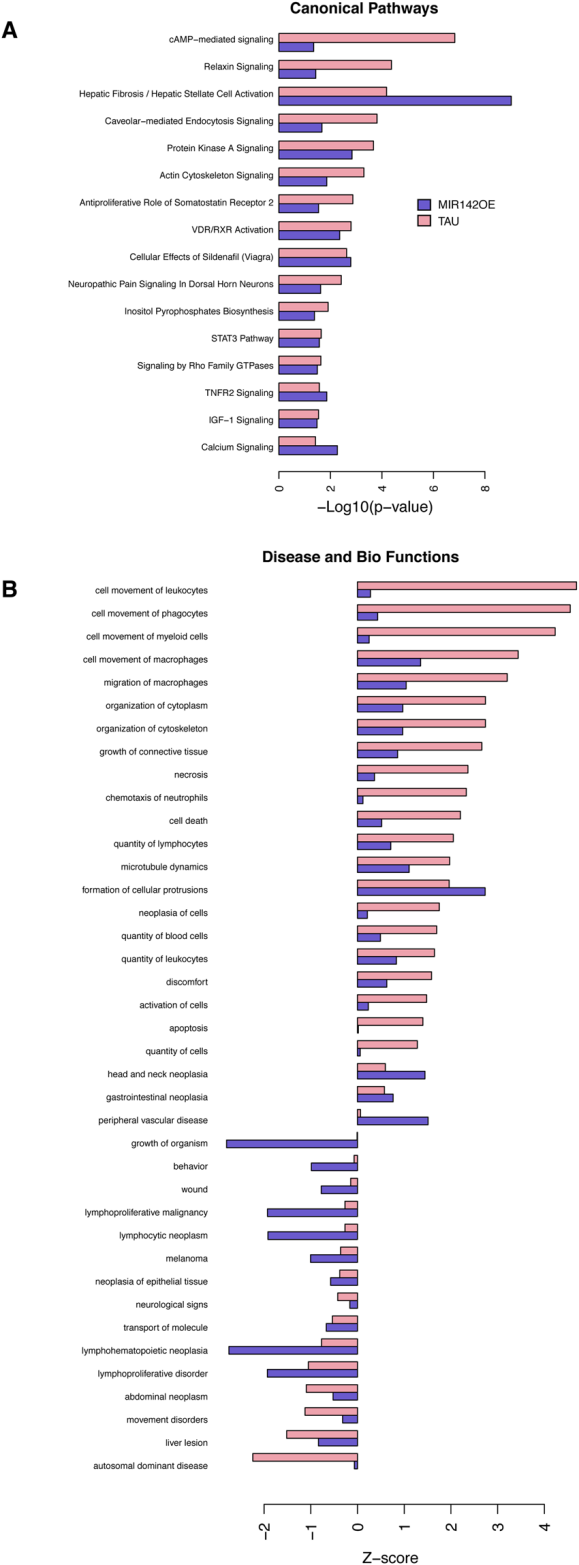
Gene network and pathway overlap between Tau and miR142-OE mice determined by IPA analysis. (**A**) Overlap of canonical pathways between Tau hippocampus and miR142-OE cortex tissue. (**B**) Overlap of Disease and Bio functions between Tau hippocampus and miR142-OE cortex tissue.

Overlapping gene expression changes between miR142 and Tau OE prompted us to examine the contributory role of miR142 to the gene networks and disease pathways caused by Tau-OE. To assess this, we compared the canonical pathways as well as disease and biological functions between miR142-OE and Tau-OE samples. Even though miR142-OE and Tau-OE models target several different genes, a high degree of directional similarity in the pathway regulation was observed between the two groups (Fig. 7). With respect to inflammatory pathways, the canonical pathways that were similarly changed include signal transducer and activator of transcription 3 (*Stat3*) pathway and tumor necrosis factor receptor 2 (*Tnfr2*) signaling (Fig. 7A). *Stat3* is a transcription factor involved in cytokine mediated signaling pathways and its activation promotes astrogliogenesis associated with brain inflammation^48^. Upon TNF binding, *Tnfr2* can activate apoptosis and inflammation^49,50^. Disease and biological functions also revealed similar and robust changes in macrophage migration, movement and phagocytosis, suggesting an increase in inflammatory signals (Fig. 7B).

## Discussion

Here we identified several miRs that are altered in both the presymptomatic and symptomatic stages of tauopathy. To explore whether miR changes can result in the gene expression alterations observed in Tau-OE, we explored the predicted targets of the differentially expressed miRs and compared the overlap between these predicted targets and the mRNA changes in 2 m Tau hippocampi. Subsequent pathway analysis of the overlapping genes identified several important signaling pathways, with neuroinflammation as the top pathway. Using the IUE surgical technique, we were able to provide the first *in vivo* demonstration that neuronal miR142-OE in wildtype cortex leads to gliosis as well as dysregulation of genes involved in several disease pathways. Many of these processes are also altered in Tau hippocampi, such as inflammation. The upregulation of *Gfap* and *Csf1* mRNA levels in miR142-OE cortex or Tau(P301L) OE hippocampi are likely to cause the increase of microglia and reactive astrocytes. Taken together, our data suggest that Tau-OE induces presymptomatic miR-changes and such miR alterations are likely to mediate alterations in gene regulatory networks, and disease and biological pathways.

Previous transcriptional analysis with mouse tauopathy models has revealed pathways in neuronal function, cell metabolism, signal transduction and stress response at symptomatic stages^51,52^. Here we found significant mRNA changes in Tau hippocampi as early as 2 m. Interestingly, the expression changes of genes such as *Gfap*, *Csf1*, *Prnp*, *C4b* and *Trem2*, identified with 2 m Tau hippocampi (Fig. 2; GSE107183) have been observed at symptomatic stages in the same rTg4510 tauopathy model in other studies^52,53^. These genes are involved in various aspects of inflammatory responses and neurodegeneration. Astrocyte reactivity, which is characterized by *Gfap* overexpression, contributes to many neurodegenerative diseases^54^. Meta-analysis of gene expression studies to identify common transcriptomic signatures for neurodegenerative diseases has identified *Csf1* as a common inflammatory marker^55^. *Prnp* has been implicated as a causative protein agent in tauopathies and many neurological disorders including AD, Parkinson’s disease, Lou Gehrig’s disease and Creutzfeldt-Jakob disease^56^. Complements are known in modulating the inflammatory axis in many neurodegenerative disorders^57,58^. Dysregulated *Trem2* signaling in microglia has been shown to be involved in both Tau and amyloid pathology, most likely by mediating microglial responses to neuroinflammation^59–61^.

Several predicted miR-regulated genes from our miR-RNA pairing analysis are involved in the inflammatory response, such as *Trem2, Csf1r, Il6r*, Phosphatidylinositol-4,5-Bisphosphate 3-Kinase Catalytic Subunit Gamma (*Pik3cg*)^62^, Ras-Related C3 Botulinum Toxin Substrate 2 (*Rac2*), and *Tgf*β receptor 1/2 (Supplementary Table 6). Aberrant expression of *Rac2* has been reported in the pathogenesis of childhood pre-B-cell acute lymphoblastic leukemia in a mouse model^63^. Enhanced *Tgf*β*1* signaling following brain injury has been shown to alter immune function in brain pericytes by attenuating the expression of several chemokines and adhesion molecules^64^. miR-mRNA pairing analysis also revealed several miR gene targets involved in Neuroinflammation, Complement System, cAMP-mediated signaling, and G-Protein Coupled Receptor (GPCR) signaling. Many complement proteins, including components of *C1q*, are important mediators of inflammatory response in several neurodegenerative disorders such as AD^65,66^, Parkinson’s disease^67^, Huntington’s disease^68^ and Prion diseases^69^. Altered cAMP-mediated signaling has been shown in many neurodegenerative diseases^70^. Many components of this pathway including cAMP, protein kinase, PKA and cyclic AMP response element-binding protein (*Creb*) are implicated in Tau phosphorylation and neurofibrillary tangles^71^ as well as cognitive decline in AD^72^. Studies have explored the involvement of GPCRs in the progression of AD^73^. Mechanisms involved in the regulation of expression, degradation and trafficking of BACE1, a key enzyme in AD pathogenesis, by GPCRs have provided insights into the pathophysiology of AD as well as development of potential therapeutic modalities^74^.

MiR142 is an evolutionary conserved pre-miR that encodes for two separate miRs, miR142-3p and miR142-5p. Both miRs are co-transcribed, and share many common targets^33,75^. However, post-transcriptional regulation may account for their differential expression levels observed in our sequencing dataset (GSE106967). Several studies have delineated the role of miR142 in immune responses in multiple pathological conditions^29,33,75–77^. We found that miR142 (−3p and −5p) induction occurs soon after Tau(P301L) OE triggered by CaMKIIα-tTA, and it persists through later stages. Many cytokines and inflammation associated molecules have been characterized as direct or indirect targets of miR142, suggesting its quintessential role in inflammatory processes^33,36^. miR142 alteration has also been associated with many pathological conditions, such as cardiomyopathy, atherosclerosis, osteoarthritis and chronic fibrosis^33,76,78,79^. A recent study highlighted the role of miR142 in multiple sclerosis (MS), a neurodegenerative disease characterized by immune system dysfunction. Here, we provide experimental evidence that neuronal miR142-OE in wildtype brains upregulates expression of many inflammatory genes accompanied by an increase of microglia and reactive astrocytes.

The miR-mediated modulation of a cohort of genes that lie upstream of signaling pathways, may lead to robust effects even with subtle changes in miR expression^33,80–82^. Similar effects were observed in our study, where modest miR142 overexpression in cortical neurons resulted in widespread transcriptome changes. Interestingly, both miR142 OE and Tau OE induced many genes involved in inflammation, including *Gfap* and *Csf1*. This was also manifested in the altered regulation at the level of gene networks and pathways. Both miR142-OE and Tau-OE induced *Stat3* and *Tnfr2* signaling pathways. In addition, disease functions involved in the migration of cell types implicated in inflammation, including macrophages were elevated. Thus, manipulating a single miR, such as miR142, can lead to profound changes in biological pathways contributing to neurodegeneration. Therefore, this study shows that the early miR changes that can occur in tauopathies have pathological consequences. The deep sequencing data sets acquired in our study will allow future investigation for the coupling of miR-changes with mRNA-changes, and an understanding of how alterations in biological pathways result in neurodegeneration.

In summary, we found many canonical and disease associated pathways that are altered at the presymptomatic stage of tauopathy. Many of these pathways could be altered by miR-changes as revealed by pairing of predicted miR targets with RNAseq-validated mRNA changes. As proof-of-concept, we show that miR142-OE in cortical neurons is sufficient to trigger changes in many gene network pathways, including inflammation. Several of these pathways are also altered in presymptomatic stages of tauopathy in Tau animal model. Thus, our data provide strong support for the early miR changes triggered by Tau(P301L) expression and how these changes may impact the disease progression by altering several key biological pathways.

## Methods

### Animals

The generation and genotyping of rTg(TauP301L)4510 (rTg4510) mice were conducted as previously described^83^. rTg4510 mice over-express the P301L mutation in 4R0N human Tau associated with FTDP-17^26^. To minimize this effect of heterogeneous phenotype due to genetic background, littermate controls were used for all the experiments. All mice were housed in standard conditions with food and water provided ad libitum and maintained on a 12 hr dark/light cycle. Animals were treated in compliance with the U.S. Department of Health and Human Services and Indiana University Bloomington Institutional Animal Care and Use Committee guidelines. All experiments were performed in accordance with relevant guidelines and regulations, and were approved by Indiana University Bloomington Institutional Animal Care and Use Committee guidelines.

### RNA sequencing and pathway analysis

Small RNA sequencing was performed by LC Sciences (Houston, Texas, USA). The quantity and purity of total RNAs were assessed using a NanoDrop ND-1000 spectrophotometer (NanoDrop Inc., Wilmington, DE, USA) at a 260/280 ratio 2.0. The integrity of total RNAs was analyzed using an Agilent 2100 Bioanalyzer system and RNA 6000 Nano LabChip Kit (Agilent Tech, Santa Clara, CA, USA) with RNA integrity number greater than 7.0. The libraries were constructed from total RNA using the Illumina Truseq Small RNA Sample Preparation Protocol #15004197 Rev. F (Catalog # RS-200-9002DOC; Illumina, San Diego, CA, USA) according to the manufacturer’s protocol. Briefly, RNA adapters were ligated to target miRNAs in two separate steps. Reverse transcription reaction was applied to the ligation products to create single stranded cDNA. The cDNA was amplified by PCR using a common primer and a primer containing the index sequence and subsequently purified by PAGE-gel. Before sequencing, libraries were qualified on the Bioanalyzer High Sensitivity DNA Chip. Finally, Illumina sequencing technology was employed to sequence these prepared libraries. The raw sequences were generated using the Illumina HiSeq 2500 platform (50 bp SE, rapid-run mode). After masking of the adaptor sequences and removal of contaminated reads, the clean reads were filtered and analyzed for miRNA prediction with the software package ACGT101-miR-v4.2 (LC Sciences, Houston, Texas, USA)^84^. The data discussed here have been deposited in NCBI’s Gene Expression Omnibus^85^ and are accessible through GEO Series accession number GSE106967 (https://www.ncbi.nlm.nih.gov/geo/query/acc.cgi?acc=GSE106967).

Standard data analysis was performed on the deep sequencing by using a proprietary pipeline script, ACGT101-miR v4.2 (LC Sciences Inc.). For 2 m hippocampi, miRNA seq yielded 74,720,901 raw sequences. Of these, the total mappable sequences were 64,833,130. 67.8% (43,975,586 reads) reads mapped to the mouse miRs annotated in miRBase repository, a comprehensive database for miR sequence data^86^. The mappable reads to known mouse miRs corresponded to 1,690 miRs (see Supplementary Fig. 1 for the length distribution of mappable reads). The majority of sequences were in the range of 18–23 nucleotides, consistent with the reported length of metazoan miRs^1^. For 6 m hippocampi, sequencing of small RNA yielded 71,431,722 raw sequences. Among these, there were 64,337,423 mappable reads. The mappable reads to known miRs correspond to 1,743 mouse miRs.

mRNA sequencing was performed by Center for Medical Genomics, Indiana University School of Medicine. 1. Library Preparation and sequencing: The concentration and quality of total RNA samples was first assessed using Agilent 2100 Bioanalyzer. A RIN (RNA Integrity Number) of five or higher was required to pass the quality control. Then five hundred nanograms of RNA per sample were used to prepared single-indexed strand-specific cDNA library using TruSeq RNA Access Library Prep Kit (Illumina). The resulting libraries were assessed for its quantity and size distribution using Qubit and Agilent 2100 Bioanalyzer. One and a half pico molar pooled libraries were sequenced with 1.75 bp single-end configuration on NextSeq500 (Illumina) using NextSeq 500/550 High Output Kit. A Phred quality score (Q score) was used to measure the quality of sequencing. More than 90% of the sequencing reads reached Q30 (99.9% base call accuracy). 2. Sequence alignment and gene counts: The sequencing data were first assessed using FastQC (Babraham Bioinformatics, Cambridge, UK) for quality control. Then all sequenced libraries were mapped to the mouse genome (UCSC mm10) using STAR RNA-seq aligner^87^ with the following parameter: “–outSAMmapqUnique 60”. The reads distribution across the genome was assessed using bamutils (from ngsutils)^88,89^. Uniquely mapped sequencing reads were assigned to mm10 annotated genes using featureCounts (from subread)^89^ with the following parameters: “–s 2 –Q 10”. Quality control of sequencing and mapping results was summarized using MultiQC^90^. Genes with read count per million (CPM) > 0.5 in more than 3 of the samples were retained. The data was normalized using TMM (trimmed mean of M values) method. Differential expression analysis was performed using edgeR^91,92^. False discovery rate (FDR) was computed from p-values using the Benjamini-Hochberg procedure. The mRNA-seq data discussed here have been deposited in NCBI’s Gene Expression Omnibus^85^ and are accessible through GEO Series accession numbers: GSE107183, GSE107167 (https://www.ncbi.nlm.nih.gov/geo/query/acc.cgi?acc=GSE107183; https://www.ncbi.nlm.nih.gov/geo/query/acc.cgi?acc=GSE107167).

The multi-dimensional scaling (MDS) plot of the RNA samples was drawn using plotMDS function in edgeR. The distance represented the leading log-fold-changes between each pair of RNA samples, which is the average (root-mean-square) of the largest absolute log-fold changes between each pair^93^. This visualizes the differences between the expression profiles of different samples in two dimensions. Pathway analysis: Ingenuity Pathway Analysis (IPA, Qiagen, Germantown, MD) was performed for differentially expressed genes with FDR < 0.05. Enrichment of Canonical Pathways and Disease and Bio Functions were identified with threshold *p* < 0.05. The activity of pathways and functions was inferred as z-scores. Positive z-score indicated increased activity, while negative z-score indicated inhibited activity.

The miRNA target filter in Qiagen’s IPA software was used for pairing of the miRNA-seq and mRNA-seq data sets. Differentially expressed miRNAs (P < 0.05) were uploaded into IPA and analyzed with the miRNA target filter, which includes experimentally validated and predicted mRNA targets from TargetScan, TarBase, miRecords and the Ingenuity Knowledge Base. Differentially expressed mRNAs (FDR < 0.05) were then uploaded with the “add/replace mRNA data set” function. Using the “expression-pairing” feature, only potential targets differentially expressed in the mRNA-seq data are maintained; all other potential targets are filtered out. These target genes were then subjected to pathway analysis.

### In utero electroporation (IUE) procedure

IUE was performed as previously described^37,38^. Briefly, pregnant ICR female dams 14.5 d postgestation were anesthetized by isoflurane inhalation, and a small incision was made in the abdominal wall to expose the uterine horns. For miR142-OE overexpression experiment, a DNA mixture of pCAG-mGFP (membrane targeted green fluorescent protein [GFP]; a gift from Dr. Matt Rasband), and miRNASelect™ pEGP-mmu-mir-142 Expression Vector (Cell Biolabs) mixed in equal amounts (1ug/ul each), with a final concentration of 1 μg/μl each) into the left hemisphere of ~50% of the ICR embryos. The remaining embryos received only tdTomato (pAAV-CAG-tdTomato (codon diversified) a gift from Edward Boyden (Addgene plasmid # 59462), 1 μg/μL) to serve as littermate controls. Approximately 0.5–1 μL of DNA solution was injected into the lateral ventricle of embryos (E14.5) using a pulled glass micropipette. Each embryo within the uterus was placed between platinum tweezer-type electrodes (5 mm diameter, Harvard Apparatus, Inc., Holliston, MA, USA). Square electric pulses (33–35 V, 50 ms duration, five times) were passed at 1 s intervals using a platinum, 3-mm-diameter tweezer-type electrodes (Harvard Apparatus, Holliston, MA) using a BTX ECM 830 square Electroporator (Harvard Apparatus). The wall of the abdominal cavity and skin were then sutured, and the animals were allowed to develop to term and aged to 1 m. At 1 m old, control and miR142-OE mice were deeply anesthetized and decapitated to harvest brains, For RNAseq analysis, GFP- (miR142-OE) or tdTomato-positive (control) cortical tissues were dissected out from IUE mouse brains.

### Gene expression and miR assessment

Total RNA was purified from cortex and hippocampus using the miRVana RNA isolation kit according to the manufacturer’s instruction (part no. AM1560, ThermoFisher scientific, Grand Island, NY). One microgram of total RNA was reverse transcribed to cDNA using the iScript™ Advanced cDNA Synthesis Kit (#1725038, Bio-Rad, Hercules, CA). Quantitative real-time reverse transcriptase-polymerase chain reaction (QRT-PCR) was performed using PowerUp™ SYBR™ Green Master Mix (part no. A25742, ThermoFisher scientific). The PCR reaction was run in a Quant Studio 7 Flex Real Time PCR system (Applied Biosystems, Grand Island, NY). The gene expression was assessed using gene-specific primers. Gapdh was used as a reference control for normalization. The primer sequences were as follows: 1) mouse C4B: Forward 5′-GAA ATG TTA ACT TCC AGA AGG C-3′; Reverse 5′-CGT CTT CAT CTA TCA AGT CTT CC-3′; 2) mouse TREM2: Forward 5′-CAC CAT CAC TCT GAA GAA CC-3′; Reverse 5′-AAG GAG GTC TCT TGA TTC CTT-3′; 3) mouse-Prnp: Forward 5′-TAC CCT AAC CAA GTG TAC TAC AGG-3′; Reverse 5′-GCT GGA TCT TCT CCC GT-3′; 4) mouse GFAP: Forward 5′-AAA ACC GCA TCA CCA TTC CT-3′; Reverse 5′-GGC AGG GCT CCA TTT TCA ATC-3′; 5) mouse CSF1: Forward 5′-ACC CTC AGA CAT TGG ATT CT-3′; Reverse 5′ –AAG CTG CTT CTT TCA TCC A-3′; 6) mouse Gapdh: Forward 5′-TGC ACC ACC AAC TGC TTA GC-3′; Reverse 5′-GGC ATG GAC TGT GGT CAT GAG-3′.

The levels of gene expression in each sample were determined with the comparative Ct method using Quant Studio 7 Flex Real Time PCR software. For gene expression, cycling parameters were as follows: 2 min at 50 °C, 2 min at 95 °C, 40 cycles: 15 sec at 95 °C, and 1 min at 60 °C. For miR studies, total RNA was isolated using the mirVana miRNA Isolation Kit, according to the manufacturer’s instructions. For miR quantification by QRT-PCR, 10 ng of total RNA were used to prepare cDNA using TaqMan MicroRNA Reverse Transcription Kit (part no. 4366596, Life Technologies) and TaqMan specific probes as follows: miR142-3p (#4427975; assay ID: 000464); miR142-5p (#4427975; assay ID: 002248); miR181b (#4427975; assay ID: 465209_mat); miR181a (#4427975; assay ID: 000516); miR219-3p (#4427975; assay ID: 002390); miR219-5p (#4427975; assay ID: 000522); miR339-5p (#4427975; assay ID: 002257); miR1249 (#4427975; assay ID: 002868); U6 small nucleolar RNA (#4427975; assay ID: 001973). U6 was used as a reference control for normalization. Real-Time RT-PCR was performed using TaqMan Universal PCR Master Mix, No AmpErase UNG (#4324018, Life Technologies). The QRT-PCR cycling parameters were 2 min at 50 °C, 10 min at 95 °C, 40 cycles: 15 s at 95 °C, and 1 min at 60 °C. Data were analyzed using Quant Studio 7 Flex Real Time PCR system software (Applied Biosystems, Foster City, CA).

### Immunofluorescence staining and image acquisitions

At 1 m old, control and miR142-OE mice manipulated via IUE were deeply anesthetized by isoflurane inhalation, transcardially perfused with PBS, followed by 4% Paraformaldehyde (PFA) solution in PBS, after which the brains were removed and postfixed overnight in 4% PFA at 4°. The brains were serially sectioned in the coronal plane into 100 μm thick sections using a Leica VT-1000 vibrating microtome (Leica Microsystems, Bannockburn, IL). Brain slices containing comparative morphology were selected for immunofluorescence staining. For immunofluorescence staining, brain slices were washed with PBS/0.01% Triton X-100 (PBST) and permeabilized with 0.3% Triton X-100 in PBS at room temperature for 20 min. Next, blocking was performed for 1 h using 3% normal goat serum in PBST. Following this step, the cells were stained with various primary antibodies diluted in 3% normal goat serum/0.2% Triton X-100 in PBS at 4 °C overnight. The next day, cells were washed with PBST, and incubated with secondary antibodies (goat-anti rabbit/mouse/chicken IgG conjugated with Alexa 647/555/488 (1:1000 dilution in 0.2% Triton X-100 in PBS) was used to detect primary antibody at room temperature for 1 hour. Following this incubation, sections were washed with PBST three times for 10 min each, mounted in Vectashield mounting media with DAPI (Vector Labs, Burlingame, CA) and cover slipped for imaging. The primary antibodies used were: IBA1 (anti rabbit, 1:500, WAKO 019-19741, Richmond, VA); GFAP (anti rabbit, 1:500, DAKO Z0334, Santa Clara, CA); NeuN (anti mouse, 1:500, Millipore MAB377, Burlington, MA); S100B (anti mouse, 1:500, Sigma AMAb91038, St. Louis, MO); GFP (anti chicken,1:500, Aves Labs Inc GFP-1020, Tigard, OR).

Z stack images were obtained using Leica DFC365FX Microsystems Confocal microscope with 10x/0.30 objective. The Z-stacks were taken with 1 μm step between individual planes. All images were processed in Adobe Photoshop for brightness/contrast, orientation and background correction to better illustrate staining patterns.

### Dual ***in situ*** hybridization and immunostaining

DIG-label *in situ* hybridization and immunostaining was performed on floating brain sections. The animals were deeply anesthetized by isoflurane inhalation, and transcardially perfused with freshly prepared 4% Paraformaldehyde (PFA, catalog no # P6148, Sigma Aldrich, MO, USA). Day 1: 60 µM sections were cut on Leica VT-1000 vibrating microtome (Leica Microsystems, Bannockburn, IL) and treated with proteinase K solution (Catalog no # 3115887001, Roche, Indianapolis, IN) for 30 min at 37 °C. To reduce non-specific binding, acetylation step was performed by incubating the sections with acetylation solution (0.5% acetic anhydre (Sigma Aldrich), 1.35% triethanolamine (pH 8, Sigma Aldrich), 0.067% HCl (Fluka Chemika, Buchs, Switzerland) in 0.1% DEPC-treated water (Sigma Aldrich). Probes (miRCURY LNA detection control probe U6, catalog no # 99002-15, Exiqon, Germantown, MD; miRCURY LNA detection probe hsa-miR-142-5p, catalog no # 38514-15, Exiqon, Germantown, MD) at a final concentration of 30 nM were diluted in hybridization buffer (Formamide, SSC, Yeast RNA, Heparin, Denhardts solution, Tween, EDTA) and heat-denatured at 90 °C for 2 minutes after which it was cooled on ice. Sections were incubated O/N at 55 °C with the probes (300 µL) in a wet chamber humidified with 50% Formamide-5 × SSC(sodium chloride, sodium citrate) solution. Day 2: Multiple washes were performed with 5XSSC solution, followed by blocking for 1 h at RT with blocking solution (0.1% BSA, 1X PBS, 0.1%Tween). Next, sections were incubated with antibodies against DIG (Anti-Digoxigenin-AP, Fab fragments; Catalog no #11093274910, Roche, Indianapolis, IN), NeuN and IBA1 (1:500 dilution) for 2 nights at 4 °C. Day 3: Multiple washes with 1X PBS and 1X PBS + 0.3%Tween were performed at RT. Sections were incubated with appropriate fluorescent secondary antibodies (1:500 dilution) at RT for 2 hours. Sections were washed with 0.1 M Tris 8.2 for 10 min and developed at RT till a red precipitate started to appear. The color reaction was stopped with 1X PBS + 0.3%Tween and sections were transferred to 1 M Phosphate buffer (3 × 5 min.) after which they were incubated for 10 minutes in 1 μg/ml Hoechst (Invitrogen, Grand Island, NY) in 1 M PB. After rinsing in PB (2 × 5 min.) sections were mounted with prolong gold mounting media for imaging.

### Primary neuronal culture and lentivirus transduction

ICR pregnant females were deeply anesthetized by isoflurane inhalation, and a small incision was made in the abdominal wall to harvest their embryos for brains. Cortical tissue from E16.5 ICR embryonic brains was isolated and dissociated to acquire cortical neurons using the Worthington Papain Dissociation kit according to manufacturer’s protocol (Worthington Inc.). Cortices from several embryonic brains were pooled for plating. Neurons were plated at 100,000 cells per well of a 6-well plate. Cultures were maintained in a regularly replaced Neurobasal media (Invitrogen, Grand Island, NY) with B27 supplement (Invitrogen), penicillin-streptomycin supplement (Life Technologies) and L-glutamine (Invitrogen)^94^.

Neurons were incubated at 37 °C and 5% humidity. On 7^th^ day-*in-vitro* (DIV7), control (VSM5954, Dharmacon; GE Healthcare) and MiR142-3p-OE (VSM6215-213652311, Dharmacon, Lafayette, CO) lentiviruses were added at the titer concentration of 10^8^ transfection units/ml. On DIV11, total RNA was extracted using miRVana RNA isolation kit. Two independent experiments were performed from two different pregnant females and each experiment was conducted with three replicates.

### Data and Statistical analysis

Data were analyzed using GraphPad Prism 7.0 software (GraphPad Software, San Diego, CA, USA). Means were compared across groups with the use of *t* tests for 2 groups for data that followed the normal distribution. Normality was assessed with the Kolmogorov-Smirnov test and significant P values were denoted by *. When the data did not follow the normal distribution, *P* values were computed with nonparametric Mann-Whitney methods, and significant p values were denoted by #. Two-tailed *P* values < 0.05 were considered significant. Values are presented as means ± SEM.

## Electronic supplementary material

Supplementary Information

Sup. Table 4

Sup. Table 5

Sup. Table 6

Sup. Table 7

Sup. Table 8

Sup. Table 9

Sup. Table 10
