# Supplementary material for: Presymptomatic change in microRNAs modulates Tau pathology
Source: Sci Rep. 2018 Jun 18;8:9251. doi: 10.1038/s41598-018-27527-6 (PMC6006352; doi:10.1038/s41598-018-27527-6)
Supplement: Supplementary file 1 — Supplementary Information [file 41598_2018_27527_MOESM1_ESM.pdf]

Title: Presymptomatic change in microRNAs modulates Tau pathology

Authors and author affiliation: Salil Sharma[1], Ines Khadimallah[2], Adam Williamson Corya[1], Yousuf Omar Ali[1], Xi Rao[3], Yunlong Liu[3], Hui-Chen Lu[1,*]

[1]Department of Psychological and Brain Sciences, the Linda and Jack Gill Center for Bimolecular Sciences, Indiana University, Bloomington, IN 47405; [2]Department of Fundamental Neurosciences (DNF), University of Lausanne (UNIL), Lausanne, Switzerland; [3]Medical and Molecular Genetics, Indiana University School of Medicine, Indianapolis, Indiana 46202

*** Corresponding Author and Address:**
Hui-Chen Lu, Ph.D.
Department of Psychological and Brain Sciences
Indiana University
1101 E. 10th Street
Bloomington, IN 47405
Tel: 812-856-4998
Fax: 812-856-7187
Email: hclu@indiana.edu

Supplementary Fig 1

A

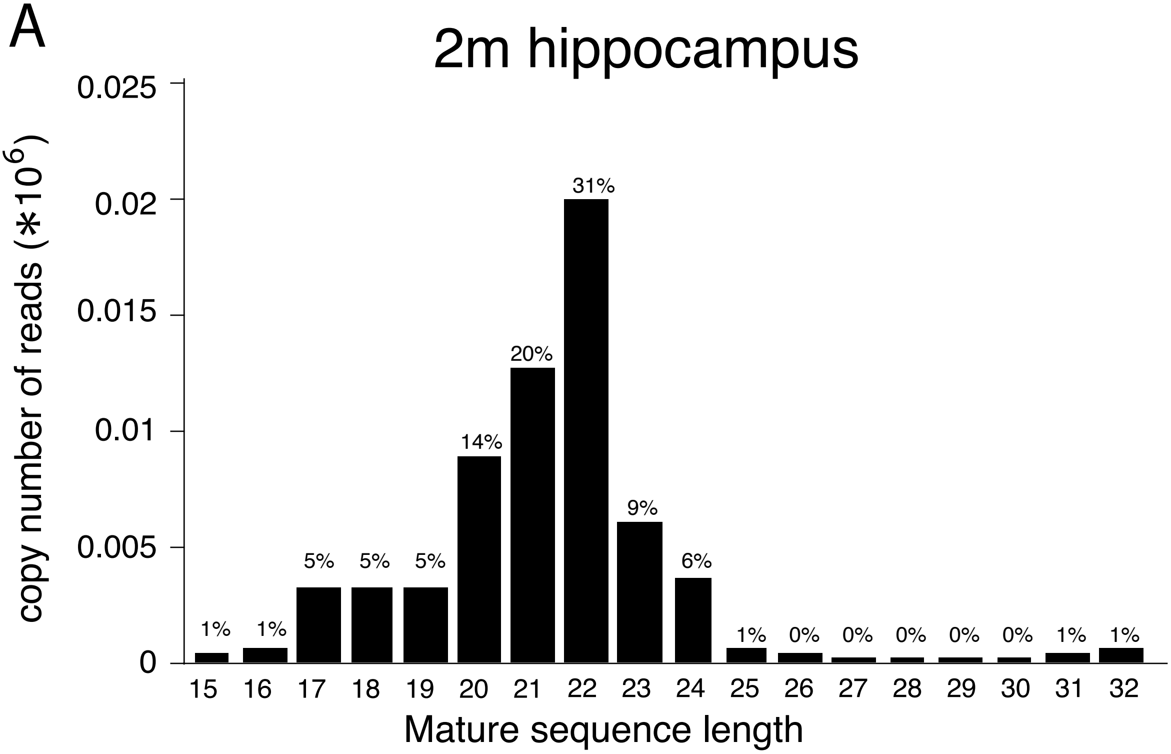

B

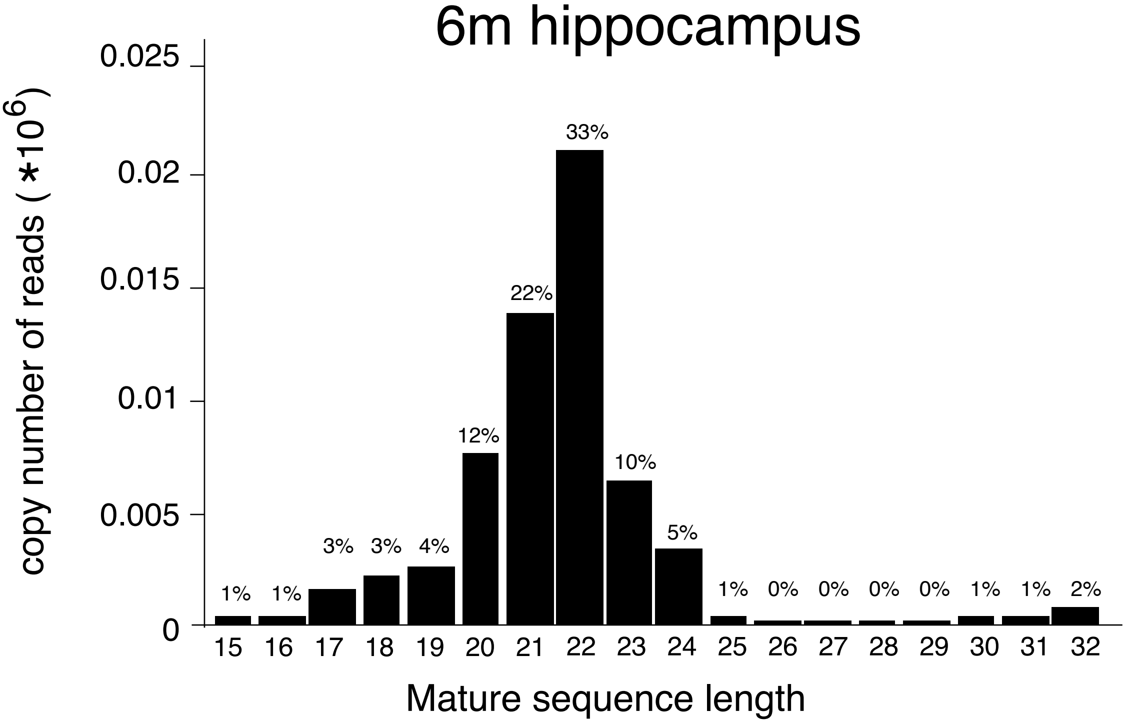

Supplementary Fig 2

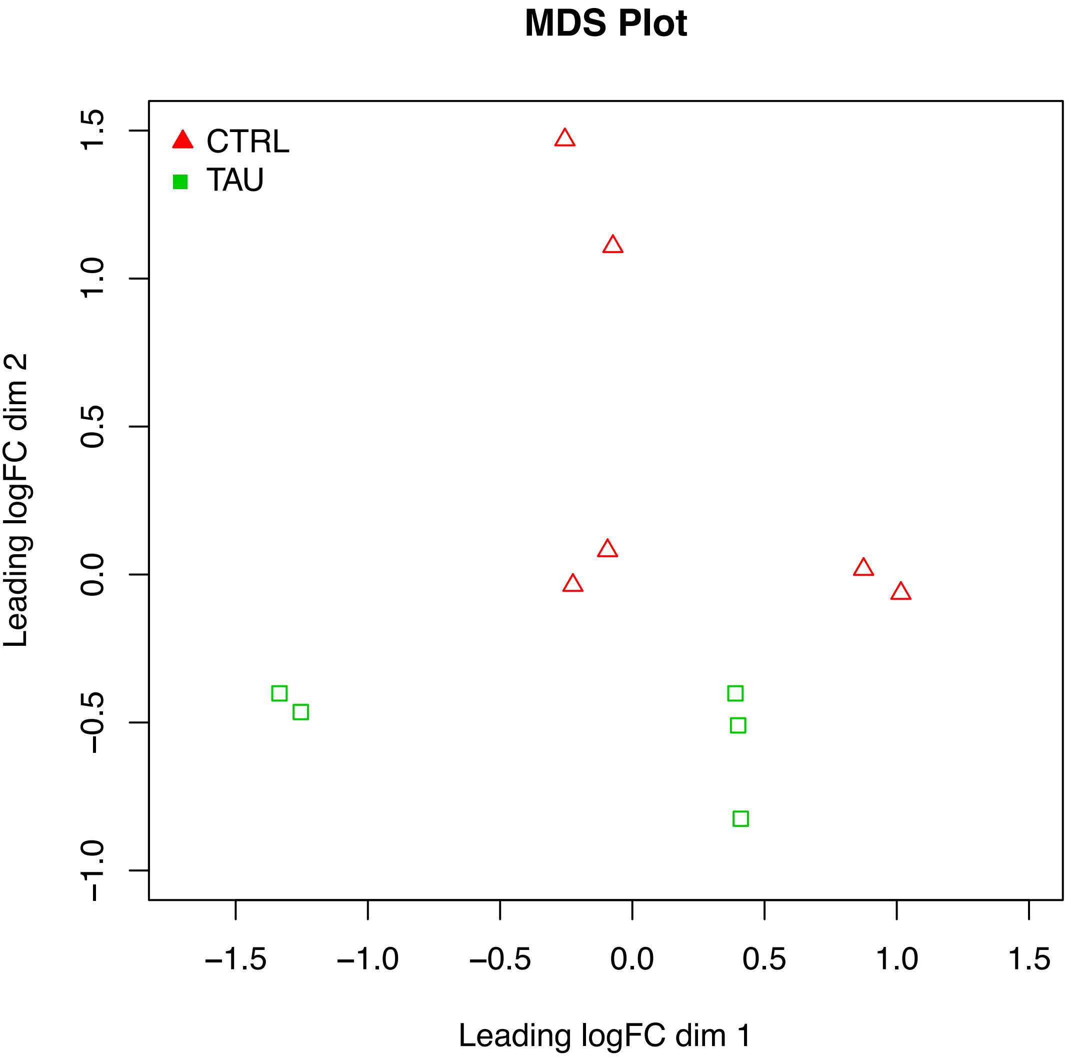

Supplementary Fig 3

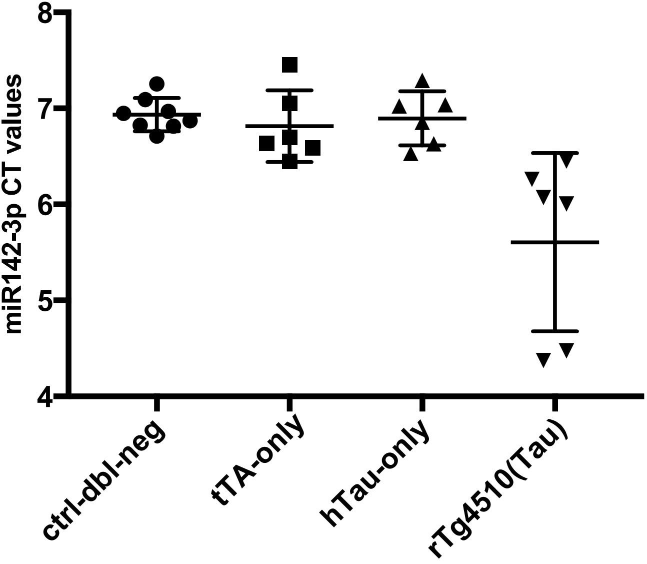

Supplementary Fig 4

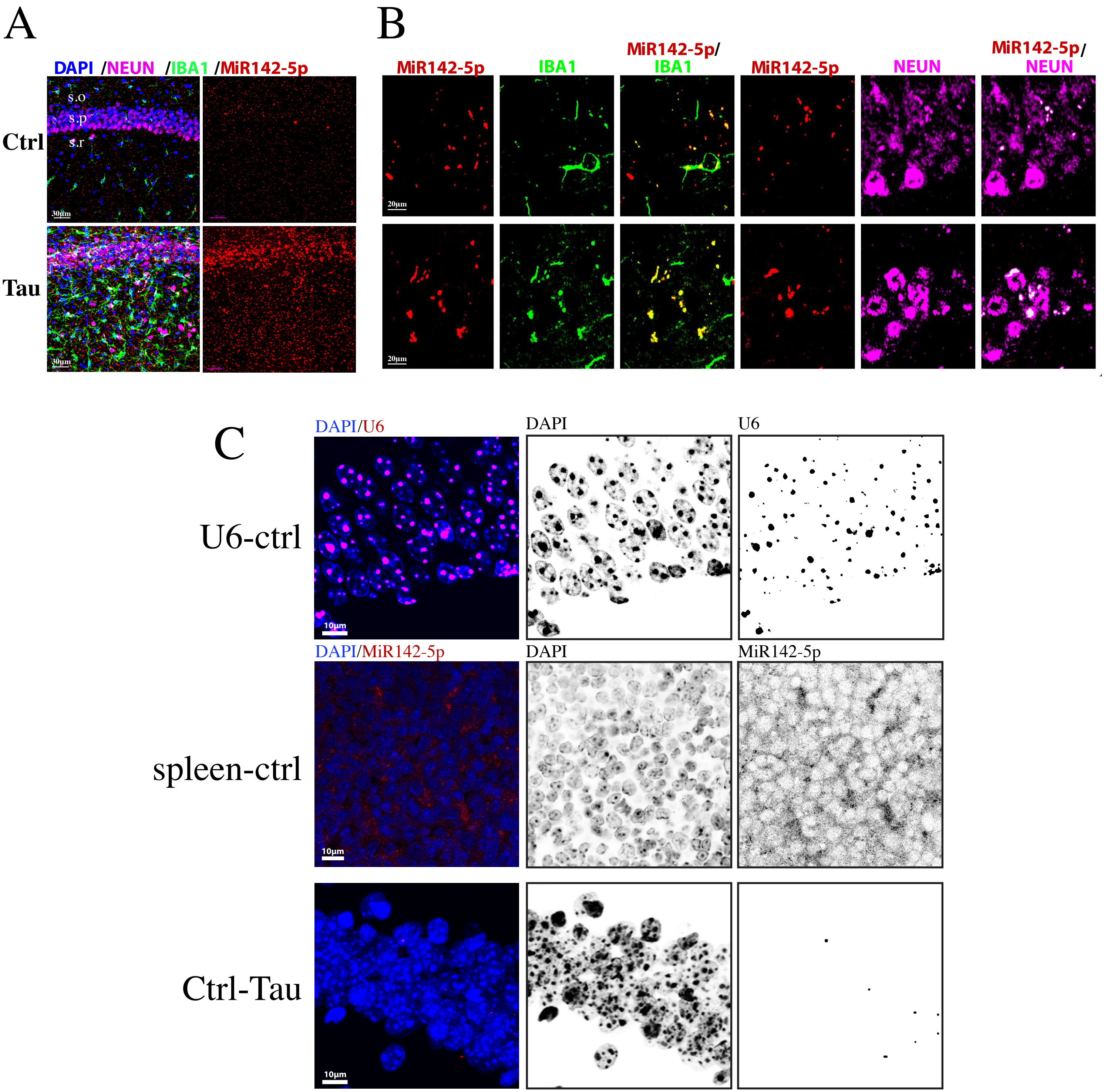

Supplementary Fig 5

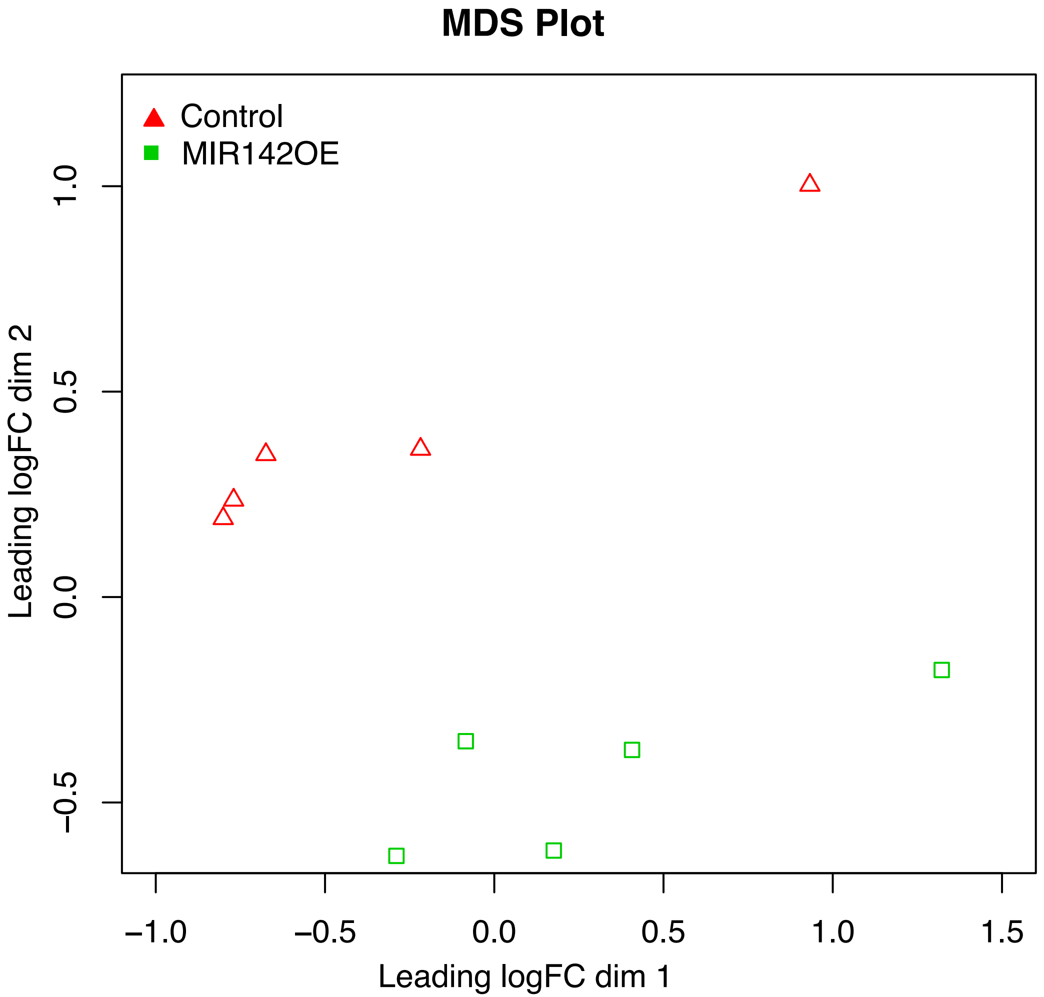

Supplementary Table 1: Hippocampus tissue information for RNA sequencing.

Age	Treatment	Tau	CAMKIIα-tTA	Sex
2M	Control1	Negative	Negative	F
	Control2	Negative	Negative	F
	Control3	Negative	Negative	F
	Tau1	Positive	Positive	F
	Tau2	Positive	Positive	F
	Tau3	Positive	Positive	F
6M	Control1	Negative	Negative	M
	Control2	Negative	Negative	M
	Tau1	Positive	Positive	M
	Tau2	Positive	Positive	M
	Tau3	Positive	Positive	M
	Tau4	Positive	Positive	M

Supplementary Table 2. Differentially regulated microRNAs at 2 months

#	Accession ID	miR Name	Ctrl-reads (mean)	Tau-reads (mean)	p-value
Reads greater than or equal to average reads (7059)					
1	MIMAT0000590	mmu-miR-342-3p	17222.9250	10925.3850	0.0030
2	MIMAT0000383	mmu-let-7d-5p	61842.8080	47847.7240	0.0070
3	MIMAT0000163	mmu-miR-153-3p	16263.8970	7721.4640	0.0110
4	MIMAT0000133	mmu-miR-101a-3p_R+1_1ss21AC	13774.8440	22129.2610	0.0130
5	MIMAT0003127	mmu-miR-484	14264.9740	9327.4350	0.0140
6	MIMAT0000673	mmu-miR-181b-5p	7049.9250	11664.6270	0.0140
7	MIMAT0000137	mmu-miR-126a-5p	14978.4730	21055.9010	0.0140
8	MIMAT0003151	mmu-miR-378a-3p_R+1	12119.2510	8760.6190	0.0190
9	MIMAT0000248	mmu-miR-30e-5p_R+2	118424.9560	157705.2710	0.0200
10	MIMAT0004746	mmu-miR-409-5p	21452.2360	9797.0180	0.0240
11	MIMAT0000674	mmu-miR-181c-5p	7346.0270	9209.5900	0.0300
12	MIMAT0000125	mmu-miR-23b-3p_R+3	6219.5360	11582.2720	0.0300
13	MIMAT0000210	mmu-miR-181a-5p	40005.2330	76645.8140	0.0300
14	MIMAT0000537	mmu-miR-27a-3p	10046.1920	15952.9280	0.0380
15	MIMAT0000677	mmu-miR-7a-5p_R+1	19582.2740	7051.2790	0.0410
16	MIMAT0000534	mmu-miR-26b-5p_R+1	22185.9100	26712.9250	0.0420
17	MIMAT0000159	mmu-miR-149-5p	8732.0630	6628.4330	0.0430
18	MIMAT0000138	mmu-miR-126a-3p_R-1	39121.6730	50870.8800	0.0440
19	MIMAT0000515	mmu-miR-30d-5p_R-2	98229.8670	129900.8150	0.0440
20	MIMAT0000546	mmu-miR-103-3p	14693.1980	11201.2170	0.0450
21	MIMAT0000678	mmu-miR-7b-5p_R+1	10355.1020	5403.6650	0.0480
22	MIMAT0022841	mmu-miR-219a-2-3p	5110.6020	10046.7080	0.0490
Reads less than average reads (7059)					
1	MIMAT0000571	mmu-miR-331-3p_R+1	1880.3688	1677.8590	0.0002
2	MIMAT0004624	mmu-miR-15a-3p_1ss22AT	64.5124	106.6957	0.0003
3	MIMAT0014808	mmu-miR-344d-3p	426.8822	247.6720	0.0009
4	MIMAT0019349	mmu-miR-101c_L+1R+2	1001.2743	1763.5193	0.0023
5	MIMAT0004826	mmu-miR-146b-3p	38.6537	57.6857	0.0040
6	MIMAT0017018	mmu-miR-16-2-3p_R-1	26.3185	42.4596	0.0042
7	MIMAT0016997	mmu-miR-187-5p	270.8221	59.9853	0.0043
8	MIMAT0005291	mmu-miR-582-5p	1897.3794	3285.7356	0.0044
9	MIMAT0017040	mmu-miR-350-5p_R+2	42.6725	95.2445	0.0049
10	MIMAT0005292	mmu-miR-582-3p_L-1R+2	776.1475	1397.9157	0.0058
11	MIMAT0003730	mmu-miR-592-5p_L-1	458.5517	686.8648	0.0059
12	MIMAT0014823	mmu-miR-3057-3p	21.5805	11.6656	0.0064
13	MIMAT0006942	oan-miR-103-3p_R+1	59.5052	38.6641	0.0066
14	MIMAT0017068	mmu-miR-181c-3p	1132.1045	1571.3509	0.0070

15	MIMAT0001094	mmu-miR-412-3p_L+1	58.4771	32.4741	0.0079
16	MIMAT0003735	mmu-miR-672-5p	572.7475	320.9741	0.0081
17	MIMAT0003711	mmu-miR-652-3p_R+1	5872.0751	2566.8260	0.0102
18	MIMAT0004684	mmu-miR-362-3p_1ss22AT	272.8543	337.9687	0.0102
19	MIMAT0003507	mmu-miR-500-3p_R-1	387.9997	297.3728	0.0110
20	MIMAT0000216	mmu-miR-187-3p_R+1	3112.5654	683.4170	0.0113
21	MIMAT0017209	mmu-miR-541-3p	317.3998	100.0163	0.0122
22	MIMAT0000209	mmu-miR-129-5p	2494.5915	2094.7373	0.0124
23	MIMAT0000711	mmu-miR-365-3p	1577.8789	994.9661	0.0126
24	MIMAT0000152	mmu-miR-140-3p_L-1R+2	4802.0084	5788.5101	0.0127
25	MIMAT0005792	hsa-miR-320b_2ss20CA22AT	47.0132	27.9594	0.0136
26	MIMAT0003898	mmu-miR-760-3p_R+2	747.3670	331.1073	0.0139
27	MIMAT0019339	mmu-miR-28c_L+1	359.5167	269.7986	0.0139
28	MIMAT0010560	mmu-miR-1249-3p	1133.8087	1931.7656	0.0141
29	MIMAT0005859	mmu-miR-1198-5p	754.4421	550.4306	0.0142
30	MIMAT0000666	mmu-miR-320-3p	3914.0798	2634.4592	0.0161
31	MIMAT0027807	mmu-miR-6953-3p_R-1	13.2084	7.2927	0.0165
32	MIMAT0003894	mmu-miR-764-5p_R+3	46.7483	17.3068	0.0167
33	MIMAT0036137	chi-miR-326-5p_1ss5GA	25.4076	43.8821	0.0170
34	MIMAT0017017	mmu-let-7f-2-3p_R+1	230.4440	291.2281	0.0178
35	MIMAT0035718	mmu-miR-935_L-2	162.3808	127.1211	0.0185
36	MIMAT0025585	mmu-miR-6540-5p_R-1	438.9607	273.1388	0.0187
37	MIMAT0016992	mmu-miR-153-5p_L+5	576.2505	366.5502	0.0188
38	MIMAT0000584	mmu-miR-339-5p_R-2_1ss21AT	383.3649	517.9357	0.0188
39	MIMAT0014926	mmu-miR-344b-3p	567.2167	276.0596	0.0192
40	MIMAT0004528	mmu-miR-125a-3p_R-1	374.1257	262.7612	0.0195
41	MIMAT0003490	mmu-miR-700-3p	202.7094	125.7502	0.0196
42	MIMAT0000532	mmu-miR-23a-3p	3139.2190	5254.2089	0.0201
43	MIMAT0000233	mmu-miR-200b-3p	4202.5302	230.5671	0.0201
44	MIMAT0005851	mmu-miR-1193-3p	713.8030	433.9682	0.0205
45	MIMAT0000664	mmu-miR-219a-5p	1745.0204	3593.1071	0.0205
46	MIMAT0017070	mmu-miR-7a-2-3p	204.9704	86.4919	0.0209
47	MIMAT0014810	mmu-miR-1298-3p	1099.4219	424.2450	0.0210
48	MIMAT0024360	ppy-miR-378d_R+3	139.3350	74.4526	0.0211
49	MIMAT0000597	mmu-miR-346-5p	6759.8102	1540.1083	0.0212
50	MIMAT0014934	mmu-miR-3102-5p.2-5p_R-1	59.8325	27.0112	0.0213
51	MIMAT0004529	mmu-miR-125b-2-3p_R-1	4077.9593	5641.1494	0.0219
52	MIMAT0000660	mmu-miR-181a-1-3p	1343.2453	2708.9901	0.0219
53	MIMAT0004120	mdo-miR-187-3p_R+1	43.0333	10.1009	0.0225
54	MIMAT0014803	mmu-miR-1264-3p	2957.6974	587.0522	0.0226
55	MIMAT0004653	mmu-miR-342-5p_R-1	443.7011	316.8661	0.0234

56	MIMAT0014802	mmu-miR-1264-5p	259.9885	86.5706	0.0238
57	MIMAT0014877	mmu-miR-3084-3p	27.3678	44.0977	0.0246
58	MIMAT0003780	mmu-miR-490-3p_R+1	1603.9012	2775.1617	0.0253
59	MIMAT0006835	oan-miR-181b-5p_R+7	27.9923	35.7060	0.0254
60	MIMAT0003167	mmu-miR-540-3p_R+2	515.7789	449.2134	0.0258
61	MIMAT0014928	mmu-miR-344c-3p	121.3344	56.6446	0.0260
62	MIMAT0004621	mmu-let-7b-3p_1ss22CT	818.7214	1038.1058	0.0262
63		PC-5p-35674_14	7.5830	2.4778	0.0274
64	MIMAT0004889	mmu-miR-504-5p	128.7450	93.1411	0.0274
65	MIMAT0000593	mmu-miR-344-3p	866.3791	431.4883	0.0275
66	MIMAT0000236	mmu-miR-203-3p_L-1R+1	519.5805	946.8774	0.0278
67	MIMAT0004662	mmu-miR-139-3p_R+1	1749.5880	872.5954	0.0279
68	MIMAT0000154	mmu-miR-142a-5p_L+2R-3	2607.2628	3985.0584	0.0284
69	MIMAT0004821	mmu-miR-671-3p	618.7329	1008.5249	0.0289
70	MIMAT0017328	mmu-miR-1188-3p_R-2	10.5112	3.4010	0.0307
71	MIMAT0025123	mmu-let-7j_R-1_1ss8TG	4463.6111	3500.0757	0.0309
72	MIMAT0017075	mmu-miR-361-3p_L-1	1440.5507	1775.9864	0.0311
73	MIMAT0005440	mmu-miR-24-2-5p	1586.8049	2700.3089	0.0321
74	MIMAT0009457	mmu-miR-1839-3p_R-2	270.6956	380.3431	0.0332
75	MIMAT0017060	mmu-miR-221-5p	2599.4072	1688.9570	0.0339
76		mmu-mir-149-p3	7.5971	15.3336	0.0341
77	MIMAT0001537	mmu-miR-429-3p	1058.8594	87.7023	0.0345
78	MIMAT0000706	mmu-miR-362-5p	707.6584	281.3269	0.0348
79	MIMAT0000540	mmu-miR-93-5p_R-2	1263.0649	1792.8912	0.0351
80	MIMAT0000526	mmu-miR-15a-5p_R-1	664.8703	1027.6883	0.0353
81	MIMAT0016916	hsa-miR-4286_R+1	5.7932	11.6903	0.0354
82	MIMAT0004629	mmu-miR-22-5p_R-1	341.1291	502.3946	0.0371
83	MIMAT0003509	mmu-miR-501-3p_R+1	1801.6016	766.7501	0.0388
84	MIMAT0000155	mmu-miR-142a-3p_R-1	124.0647	194.0203	0.0392
85	MIMAT0004644	mmu-miR-337-5p	2690.6416	2158.3830	0.0408
86	MIMAT0022370	mmu-miR-5621-3p	74.2179	43.9856	0.0414
87	MIMAT0003734	mmu-miR-667-3p	2029.4411	1421.8106	0.0420
88	MIMAT0014944	mmu-miR-486b-3p_R+1	25.9110	15.3719	0.0421
89	MIMAT0004656	mmu-miR-345-3p_L+1R-1_1ss22AT	483.7142	328.6830	0.0432
90	MIMAT0022384	mmu-miR-344h-3p	8.4994	2.8570	0.0438
91	MIMAT0023746	cgr-miR-1260_R+3	102.8034	174.8222	0.0446
92	MIMAT0000612	mmu-miR-135b-5p	514.4915	293.0913	0.0451
93	MIMAT0000519	mmu-miR-200a-3p	6293.9471	442.8763	0.0458
94	MIMAT0017069	mmu-miR-128-2-5p	334.8365	191.1010	0.0467
95	MIMAT0017282	mmu-miR-544-5p	957.8717	623.3711	0.0480
96	MIMAT0017067	mmu-miR-181b-1-3p_R+2	69.8928	133.0907	0.0484

97	MIMAT0000160	mmu-miR-150-5p	4439.6832	6448.1987	0.0485
98	MIMAT0004820	mmu-miR-744-3p	155.2825	262.7418	0.0486

Supplementary Table 3. Differentially regulated microRNAs at 6 months

#	Accession ID	Name	Ctrl-reads (mean)	Tau-reads (mean)	p-value
Reads greater than or equal to average reads (7616)					
1	mmu-miR-221-3p	MIMAT0000669	20697.0118	7951.0457	0.0058
2	mmu-miR-143-3p_R+1	MIMAT0000247	140672.4247	92946.7573	0.0088
3	mmu-miR-338-3p_R-1	MIMAT0000582	11771.9859	22578.0830	0.0112
4	mmu-miR-219a-2-3p	MIMAT0022841	5764.2987	9115.6537	0.0131
5	mmu-miR-221-5p_R-4	MIMAT0017060	7648.3178	1828.4888	0.0167
6	mmu-miR-103-3p	MIMAT0000546	15123.6637	9885.3936	0.0210
7	mmu-miR-744-5p_R-1	MIMAT0004187	13593.5815	7321.5200	0.0224
8	mmu-miR-26b-5p_R+1	MIMAT0000534	22637.1354	30484.1688	0.0272
9	mmu-miR-222-3p_R+2	MIMAT0000670	22167.7190	8430.5095	0.0316
10	mmu-miR-26a-5p	MIMAT0000533	513205.9107	428165.3741	0.0323
11	mmu-let-7d-5p	MIMAT0000383	81525.0144	50892.8572	0.0402
12	mmu-miR-9-5p	MIMAT0000142	88155.5092	59575.7830	0.0405
13	mmu-miR-378a-3p_R+1	MIMAT0003151	16043.4259	8933.7047	0.0443
14	mmu-miR-330-3p_L-1	MIMAT0000569	7623.2540	3729.2710	0.0490
Reads less than average reads (7616)					
1	mmu-miR-107-3p_R-1	MIMAT0000647	2906.1178	2349.9838	0.0018
2	mmu-miR-664-3p_R-1	MIMAT0012774	1761.7548	1448.2006	0.0024
3	mmu-miR-155-5p	MIMAT0000165	15.6553	89.1600	0.0039
4	mmu-miR-8114	MIMAT0031420	25.4082	47.9355	0.0042
5	mmu-miR-142a-5p_L+2R-3	MIMAT0000154	3604.1938	6352.3171	0.0052
6	mmu-miR-216b-5p	MIMAT0003729	3.9138	15.9094	0.0055
7	mmu-miR-181b-1-3p_R+2	MIMAT0017067	81.2203	103.0799	0.0057
8	mmu-miR-7021-5p_L+2R-2	MIMAT0027946	10.3621	5.1722	0.0059
9	cgr-miR-1260_R+2	MIMAT0023746	130.0382	214.9477	0.0065
10	mmu-miR-125b-1-3p	MIMAT0004669	1433.0540	2253.7315	0.0067
11	mmu-miR-7047-3p	MIMAT0027999	52.3078	37.0068	0.0077
12	mmu-miR-130b-3p	MIMAT0000387	27.2286	49.1028	0.0080
13	mmu-miR-141-3p_R-1	MIMAT0000153	10.2573	75.7050	0.0080
14	PC-3p-26288_26		2.1494	8.8522	0.0090
15	mmu-miR-7235-3p_R-1	MIMAT0028439	19.6812	11.5262	0.0092
16	mmu-miR-181a-1-3p	MIMAT0000660	1993.9888	2240.8242	0.0093
17	mmu-miR-669o-3p_R-1	MIMAT0017347	9.5920	12.8881	0.0097
18	PC-5p-7215_175		24.8939	79.1250	0.0111
19	mmu-miR-7226-3p_R-2	MIMAT0028421	3.9138	8.8405	0.0116
20	rno-miR-1843b-3p_1ss3TC	MIMAT0035731	350.6473	272.0225	0.0116
21	mmu-miR-25-5p_R+1	MIMAT0017049	31.1985	20.0083	0.0120
22	mmu-miR-29b-2-5p_R-2	MIMAT0017063	143.1201	105.6110	0.0133

23	mmu-miR-124-5p	MIMAT0004527	155.9453	107.9933	0.0147
24	PC-3p-23856_30		19.5691	27.9148	0.0160
25	mmu-miR-6540-3p	MIMAT0025586	4.7960	13.0985	0.0169
26	mmu-miR-15a-3p_1ss22AT	MIMAT0004624	68.4602	105.0508	0.0184
27	mmu-mir-16-1-p3		93.8195	124.6812	0.0192
28	ptr-miR-92_R+2	MIMAT0002699	803.6286	992.8661	0.0204
29	ssc-mir-4335-p5		105.0150	139.7552	0.0208
30	mmu-miR-671-3p	MIMAT0004821	924.0569	692.3528	0.0217
31	tch-mir-9771b-p5_1ss14CG		28.0352	14.3503	0.0226
32	mmu-miR-676-5p	MIMAT0003781	133.7766	117.8463	0.0236
33	mmu-miR-1843b-5p_L+1R-1	MIMAT0019345	5617.6345	4353.0472	0.0237
34	hsa-miR-4792_L+1R+2_1ss10GT	MIMAT0019964	5.2931	30.7330	0.0248
35	mmu-miR-362-3p_1ss22AT	MIMAT0004684	304.3179	386.2450	0.0251
36	mmu-miR-188-3p	MIMAT0004541	27.0117	52.6916	0.0259
37	mmu-miR-337-3p_L+1R-2	MIMAT0000578	251.5418	602.0004	0.0263
38	mdo-miR-150-5p_1ss21AT	MIMAT0012748	726.8891	981.7618	0.0281
39	mmu-miR-21a-3p_R-1	MIMAT0004628	53.0146	114.2543	0.0282
40	mmu-miR-411-3p_R-1	MIMAT0001093	410.0126	466.9449	0.0287
41	efu-mir-9277-p3_2ss17AC18GA		206.6134	345.0022	0.0299
42	mmu-miR-202-5p	MIMAT0004546	6.1753	14.9102	0.0300
43	mmu-miR-1191b-5p	MIMAT0029866	4.6839	10.8738	0.0315
44	mmu-miR-195a-5p	MIMAT0000225	4275.6671	2764.2947	0.0320
45	mmu-miR-328-3p	MIMAT0000565	6894.2386	5161.2803	0.0321
46	mmu-miR-193a-3p	MIMAT0000223	7.5547	23.6816	0.0330
47	mdo-mir-7311-p5_1ss8CG		10.6351	26.3714	0.0341
48	mmu-miR-219a-5p	MIMAT0000664	1856.5034	4047.0971	0.0348
49	mmu-miR-7044-3p	MIMAT0027993	15.7041	5.8071	0.0350
50	mmu-miR-28c_L+1R+1	MIMAT0019339	37.5045	28.8722	0.0366
51	oan-miR-1386_L-1R-1	MIMAT0007162	19.8421	34.5650	0.0368
52	mmu-miR-30c-1-3p	MIMAT0004616	164.9091	137.7552	0.0370
53	mmu-miR-182-3p_L-1	MIMAT0016995	1.7644	6.6716	0.0372
54	mmu-miR-216a-5p	MIMAT0000662	30.7990	110.6161	0.0397
55	mmu-miR-1839-5p	MIMAT0009456	3565.2743	2244.8241	0.0397
56	PC-3p-6216_220		11.3076	67.6414	0.0405
57	mmu-miR-676-3p	MIMAT0003782	2038.1113	1454.7540	0.0415
58	mmu-miR-7a-1-3p	MIMAT0004670	626.8949	479.2330	0.0415
59	mmu-miR-93-5p_R-2	MIMAT0000540	1422.9805	2062.6838	0.0423
60	mmu-miR-504-5p	MIMAT0004889	206.0330	113.5930	0.0429
61	mmu-miR-150-5p_R-1	MIMAT0000160	3439.2879	6305.0996	0.0439

62	chi-miR-30a-3p_L-1R+2	MIMAT0036122	17.5317	27.4896	0.0439
63	miR-1249-3p	MIMAT0010560	1807.8838	2515.4197	0.0445
64	mmu-miR-5129-5p_R-1_1ss12TC	MIMAT0020640	14.2271	4.0839	0.0446
65	mmu-miR-142a-3p_R-1	MIMAT0000155	137.5440	303.5018	0.0447
66	mmu-miR-139-3p_R+1	MIMAT0004662	1100.7157	678.1925	0.0448
67	efu-mir-127-p5_1ss11CG		5.6782	8.6156	0.0452
68	rno-mir-6320-p3_1ss15AC		98.9373	44.4195	0.0454
69	mmu-miR-344d-3-5p	MIMAT0014807	352.5488	126.0435	0.0460
70	mmu-miR-153-5p_L+5	MIMAT0016992	439.8190	337.1094	0.0461
71	mmu-miR-195a-3p	MIMAT0017000	89.5207	59.8487	0.0465
72	mmu-miR-450a-5p	MIMAT0001546	489.0501	373.8491	0.0466
73	mmu-miR-351-5p_R-1	MIMAT0000609	1900.5414	1269.7919	0.0468
74	mmu-miR-412-3p_L+1	MIMAT0001094	49.7733	28.1056	0.0470
75	mmu-miR-551b-5p	MIMAT0017236	67.6557	34.6577	0.0476
76	mmu-miR-1843a-3p_R-1	MIMAT0014806	132.8384	119.0427	0.0481
77	PC-3p-12459_77		11.6294	18.0646	0.0486
78	mmu-miR-299b-3p_R+4	MIMAT0022837	163.5641	140.8624	0.0499

Supplementary Fig 1: Read distribution of small RNA sequencing of (A) 2m and (B) 6m Tau hippocampi.

Supplementary Fig 2: The multi-dimensional scaling (MDS) plot shows differences among 2m control and Tau hippocampi.

Supplementary Fig 3: Relative miR142-3p expression levels in control double negative, tTA only, hTau only and rTg4510 (Tau) 2m hippocampi.

Supplementary Fig 4: (A,B) Dual ISH and IHC staining of (A) miR142-5p (red) in microglia (green) and neurons (megenta) in the brain of 6m old Tau mice. MiR142-5p was more abundant in 6m Tau hippocampi compared to control. Strong miR142-5p signals could be detected in both $NeuN^{+}$ neurons and Iba-1^{+} microglia in Tau hippocampi CA1 area. (C) The specificity of this experiment was verified with two positive controls: U6, a noncoding snRNA, was used as a positive control for all tissues because it is present in most cell types while spleen was used as the positive control for its high levels of miR142-5p. 6m control hippocampus expresses low level of miR142-5p. s.o (stratum oriens), s.p (stratum pyramidale), s.r (stratum radiatum). Experiments were conducted with coronal brain sections prepared from 3 Tau animals with 3 littermate controls.

Supplementary Fig 5: The multi-dimensional scaling (MDS) plot shows differences among tdTomato control and miR142-OE cortex samples in 1m wildtype mice.

Supplementary Table 1: Genotype information for hippocampi used for deep sequencing.

Supplementary Table 2: List of differentially regulated miRs in 2m Tau hippocampi by miRNA sequencing.

Supplementary Table 3: List of differentially regulated miRs in 6m Tau hippocampi by miRNA sequencing.

Supplementary Table 4: List of differentially regulated genes in 2m Tau hippocampi by mRNA sequencing.

Supplementary Table 5: Canonical pathway, Transcriptomic network, and Diseases and Biofunction analysis of differentially regulated genes in 2m Tau hippocampi.

Supplementary Table 6: Overlap of miR predicted gene targets with mRNA expression in 2M Tau mice.

Supplementary Table 7: Canonical pathway, Transcriptomic network, and Diseases and Biofunctions analysis on miR-regulated gene targets in the hippocampus tissue of 2-month old rTG4510 mice

Supplementary Table 8: List of differentially regulated genes between tdtomato (ctrl) or miR142-OE cortex by RNA deep sequencing.

Supplementary Table 9: Transcriptomic network analysis of differentially regulated gene transcripts induced by miR142-OE in wildtype cortex.

Supplementary Table 10: Canonical pathway, and Diseases and Biofunctions analysis of differentially regulated gene transcripts induced by miR142-OE in wildtype cortex.
